# Musashi-2 (MSI2) regulates epidermal growth factor receptor (EGFR) expression and response to EGFR inhibitors in EGFR-mutated non-small cell lung cancer (NSCLC)

**DOI:** 10.1038/s41389-021-00317-y

**Published:** 2021-03-15

**Authors:** Petr Makhov, Igor Bychkov, Bulat Faezov, Alexander Deneka, Alexander Kudinov, Emmanuelle Nicolas, Rohan Brebion, Eleanor Avril, Kathy Q. Cai, Leonid V. Kharin, Mark Voloshin, Elena Frantsiyants, Nikolay Karnaukhov, Oleg I. Kit, Iuliia Topchu, Rushaniya Fazliyeva, Anna S. Nikonova, Ilya G. Serebriiskii, Hossein Borghaei, Martin Edelman, Essel Dulaimi, Erica A. Golemis, Yanis Boumber

**Affiliations:** 1 Molecular Therapeutics Program, Fox Chase Cancer Center, 333 Cottman Avenue, Philadelphia, PA 19111 USA; 2 Department of Medical Biophysics, N. I. Pirogov Russian National Research Medical University, Ostrovitianov Street 1, Moscow, 117997 Russia; 3 Institute of Fundamental Medicine and Biology, Kazan (Volga Region) Federal University, ul. Karl Marx 18, Kazan, 420012 Russia; 4 Department of Internal Medicine, University of New Mexico School of Medicine, 2425 Camino de Salud, Albuquerque, NM 87106 USA; 5 Temple University School of Medicine, 3500N. Broad St, Philadelphia, PA 19140 USA; 6 Histopathology Facility, Fox Chase Cancer Center, 333 Cottman Avenue, Philadelphia, PA 19111 USA; 7 National Medical research center for Oncology, 63, 14th Liniya, Rostov-on-Don, 344019 Russia; 8 Rostov State Medical University, 29, Nakhichevanskii pr, Rostov-on-Don, 344022 Russia; 9 Department of Hematology/Oncology, Fox Chase Cancer Center, 333 Cottman Avenue, Philadelphia, PA 19111 USA; 10 Department of Pathology, Fox Chase Cancer Center, 333 Cottman Avenue, Philadelphia, PA 19111 USA; 11 Present Address: Division of Hematology/Oncology, Section of Thoracic Head and Neck Medical Oncology, Robert H Lurie Comprehensive Cancer Center, Feinberg School of Medicine, Northwestern University, Chicago, IL 60611 USA

**Keywords:** Non-small-cell lung cancer, Post-translational modifications

## Abstract

Non-small cell lung cancer (NSCLC) has limited treatment options. Expression of the RNA-binding protein (RBP) Musashi-2 (MSI2) is elevated in a subset of non-small cell lung cancer (NSCLC) tumors upon progression, and drives NSCLC metastasis. We evaluated the mechanism of MSI2 action in NSCLC to gain therapeutically useful insights. Reverse phase protein array (RPPA) analysis of MSI2-depleted versus control *Kras*^*LA1/+*^*; Trp53*^*R172HΔG/+*^ NSCLC cell lines identified EGFR as a MSI2-regulated protein. MSI2 control of EGFR expression and activity in an NSCLC cell line panel was studied using RT-PCR, Western blots, and RNA immunoprecipitation. Functional consequences of MSI2 depletion were explored for cell growth and response to EGFR-targeting drugs, in vitro and in vivo. Expression relationships were validated using human tissue microarrays. MSI2 depletion significantly reduced EGFR protein expression, phosphorylation, or both. Comparison of protein and mRNA expression indicated a post-transcriptional activity of MSI2 in control of steady state levels of EGFR. RNA immunoprecipitation analysis demonstrated that MSI2 directly binds to EGFR mRNA, and sequence analysis predicted MSI2 binding sites in the murine and human EGFR mRNAs. MSI2 depletion selectively impaired cell proliferation in NSCLC cell lines with activating mutations of EGFR (EGFR^mut^). Further, depletion of MSI2 in combination with EGFR inhibitors such as erlotinib, afatinib, and osimertinib selectively reduced the growth of EGFR^mut^ NSCLC cells and xenografts. EGFR and MSI2 were significantly co-expressed in EGFR^mut^ human NSCLCs. These results define MSI2 as a direct regulator of EGFR protein expression, and suggest inhibition of MSI2 could be of clinical value in EGFR^mut^ NSCLC.

## Introduction

Non-small cell lung cancer (NSCLC) is the leading cause of cancer-related deaths in the world^1^. Eighty percent of NSCLC is non-squamous and 10–15% of these patients (~20,000/year) have disease that is characterized by an activating mutation in EGFR (EGFR^mut^)^2^, making EGFR^mut^ NSCLC one of the most common cancers and causes of cancer-related death. EGFR^mut^ disease is much more common in Asia, accounting for as much as 30–50% of NSCLC cases^2^.

In EGFR^mut^ NSCLC, tyrosine kinase inhibitors (TKIs) of EGFR have shown response rates of ~40–70%, with dramatic improvements in progression-free survival (PFS) in metastatic EGFR^mut^ NSCLC patients compared to cytotoxic chemotherapy^3^. As a result, five EGFR-targeting TKIs (gefitinib, erlotinib, afatinib, dacomitinib, and osimertinib) are FDA-approved frontline agents for patients with EGFR sensitizing mutations^3–5^. However, not all patients respond to these drugs, and virtually a majority of those that do respond will ultimately progress and die of their disease. Amongst the contributing factors that influence which tumors respond to EGFR-targeted inhibitors are the type of EGFR mutation, as well as differences in the expression level of EGFR and of additional ERBB family members (ERBB2/HER2, and ERBB3/HER3) with which EGFR can heterodimerize to signal. Given the variability of patient response to EGFR-targeted therapeutics, it is important to develop a clear understanding of which factors govern response.

Musashi-2 (MSI2) and its homolog, MSI1, are emerging as regulators of multiple critical biological processes relevant to cancer initiation, progression, stem cell compartment maintenance, and drug resistance, which are upregulated in many hematopoietic and solid tumors, including lung^6–8^. Different tumor types upregulate MSI1, MSI2, or both. Our group recently established MSI2 as upregulated in a subset of aggressive NSCLCs, and demonstrated a specific role for MSI2 in promoting metastasis in these tumors, through induction of TGFβR1 and its effector SMAD3^9^. MSI2 and MSI1 are RNA-binding proteins that regulate the stability and translation of target mRNAs encoding proteins operating in essential oncogenic signaling pathways. Besides TGFβR1/SMAD3, these targets include NUMB/Notch, PTEN/mTOR, MET, and MYC. Because Musashi regulation of targets involves physical interactions between Musashi and other proteins involved in translational control, different sets of target proteins are upregulated by Musashi in different tumor subtypes^7,10,11^.

To better understand the function of MSI2 in NSCLC, we performed a proteomic assessment of proteins in NSCLC xenografts with elevated or depleted MSI2. From this work, we found that MSI2 is essential to support the expression of EGFR in human and murine lung cancer cells, in vitro and in vivo. Through RNA-binding experiments and functional testing, we for the first time define EGFR mRNA as a direct MSI2-binding target. Importantly, we also demonstrate that EGFR^mut^ lung tumors depend on MSI2 expression, and that depletion of MSI2 enhances the sensitivity of EGFR^mut^ cells to clinical EGFR TKIs including erlotinib, afatinib, and osimertinib.

## Results

### Reverse Phase Protein Array (RPPA) analysis shows MSI2 depletion alters expression of ERBB family proteins in NSCLC cell lines

**Fig. 1:**
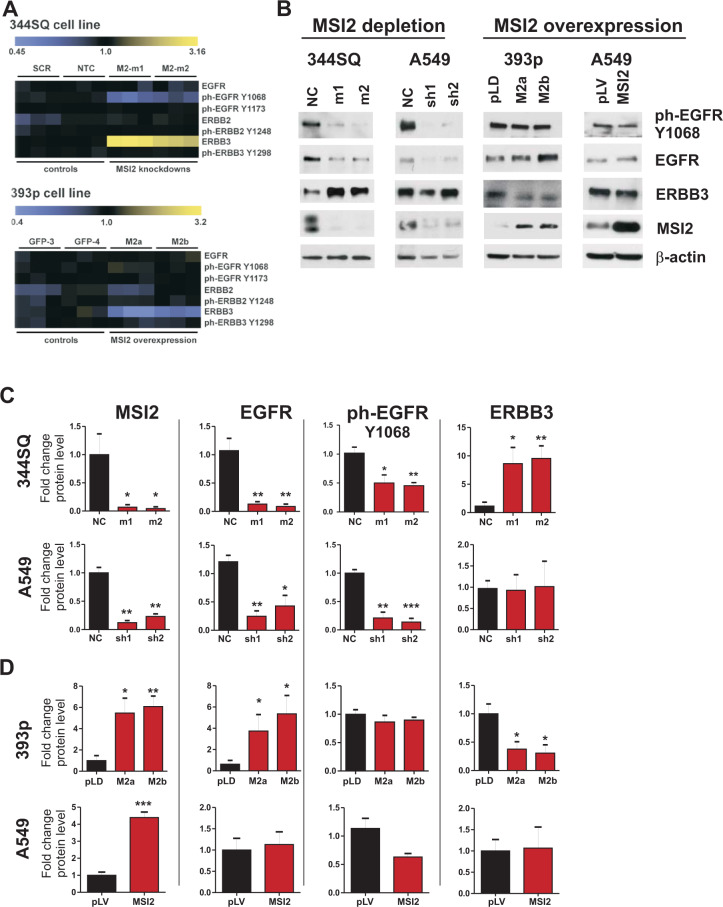
MSI2 regulation of ERBB protein expression. **A** Heatmap summarizes RPPA results for expression of EGFR, pEGFR(Y1068), pEGFR(Y1173), ERBB2, pERBB2(Y1248), ERBB3, and pERBB3 (Y1298) protein expression. Three independent isolates of cell lines were analyzed in each experiment. In stable derivatives of 344SQ, expressing high levels of endogenous MSI2, SCR, scrambled shRNA and NTC, and non-transfected cells are negative controls: M2-m1 and M2-m2 are two independent shRNAs depleting MSI2. In stable derivatives of 393p, expressing low levels of endogenous MSI2, GFP-3, and GFP-4 are negative controlsand M2a and M2b overexpress a MSI2 cDNA. **B** Western blots of indicated cell lines, following depletion (m1, m2, sh1, sh2) or overexpression (M2a, M2b, MSI2) of MSI2.NC, pLD and pLV are negative controls. MSI2 depletion was induced by the addition of 1 μg/ml of Doxycycline for 48 h. **C**, **D** Quantification of Western blot data fromat least three independent experiments by Image J software, with values normalized to β-actin. Error bars represented by SEM. Statistical analysis was performed using unpaired two tailed *t*-test. *p < 0.05, **p < 0.01, ***p < 0.001 for all graphs.

We previously identified *Kras*^*LA1/+*^*;P53*^*R172HΔG/+*^ murine NSCLC cell lines expressing high (344SQ) versus low (393P) levels of MSI2^9^. Using RPPA analysis of 171 total and phospho-proteins for expression changes, we examined protein expression changes associated with shRNA-mediated MSI2 knockdown in the 344SQ cell line, which expresses high endogenous levels of MSI2, and MSI2 overexpression in 393P cells, which has low endogenous levels^9^. MSI2 depletion in two independent derivative lines, murine 344SQ and human A549, significantly reduced expression of phospho (ph) EGFR-Y^1068^, and increased expression of the ERBB family protein ERBB3/HER3, in 344SQ cells (Fig. 1A). Reciprocally, MSI2 overexpression in 344SQ and A549 cells modestly reduced ERBB3 expression, but had no significant effect on EGFR protein expression.

### MSI2 is required for EGFR protein expression in multiple NSCLC cell lines

As homodimers and heterodimers involving EGFR, ERBB2/HER2, and ERBB3/HER3 contribute to oncogenic signaling in in NSCLC^3,9,12^, and given that some antibodies used on RPPA panels are not optimized^13^, we further investigated MSI2 control of this ERBB protein family in detail using a panel of NSCLC cell models. Western analysis of the 344SQ cell line confirmed MSI2 depletion elevated ERBB3 expression and reduced expression of phospho (ph) EGFR-Y^1068^, but also indicated MSI2 depletion significantly reduced total EGFR levels (Fig. 1B, C); there was no significant effect on ERBB2 expression (Supp Fig. S1). Conversely, overexpression of MSI2 in 393P cells increased EGFR expression, and reduced ERBB3/HER3 expression, while not affecting ERBB2/HER2 (Fig. 1B, D and Supp Fig. S1). In human KRAS/TP53-mutated cell model (A549) depletion of MSI2 strongly reduces levels of total and ph-EGFR^Y1068^. However, MSI2 overexpression did not increase EGFR expression, and neither overexpression nor depletion of MSI2 consistently affected ERBB2 or ERBB3 expression in A549 cell line (Fig. 1B–D and Supp Fig. S1).

**Fig. 2:**
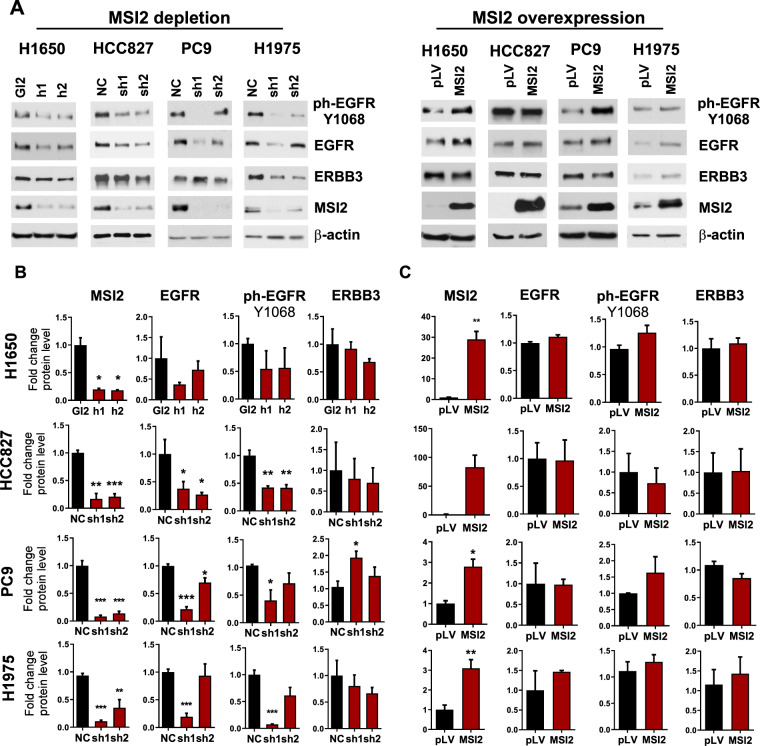
MSI2 regulation of ERBB protein expression in EGFR^mut^ cell lines. **A** Western blots of indicated cell lines, following MSI2 depletion by shRNA (sh1, sh2) or siRNA (h1, h2) or overexpression (MSI2) in three EGFR^mut^ NSCLC cell lines; H1650, HCC827, PC9, and H1975. Negative controls include GL2 and NC for depletion, and pLV for overexpression. MSI2 depletion was induced by the addition of 1 μg/ml of Doxycycline for 48 h. **B**, **C** Quantification of Western blot data from at least three independent experiments by Image J software. Error bars represented by SEM. Statistical analysis was performed using unpaired two tailed *t*-test. *p < 0.05, **p < 0.01, ***p < 0.001 for all graphs.

The biology of KRAS-mutated and EGFR-mutated (EGFR^mut^) NSCLC differs in numerous respects that might influence the role of MSI2^14^. Of particular importance would be the extent of MSI2 control of EGFR protein expression in the context of EGFR^mut^ NSCLC. We therefore investigated MSI2 control of ERBB family protein expression in four human cell models bearing activating mutations of EGFR: PC9, HCC827, H1975, and H1650 (Fig. 2 and Supp Fig. S1). In all models, MSI2 depletion consistently and strongly downregulated total and ph-^Y1068^-EGFR protein (Fig. 2A, B), but did not significantly affect expression of ERBB2/HER2 or ERBB3/HER3 (Fig. 2A, B and Supp Fig. S1). Conversely, overexpression of MSI2 had no effect on expression of any ERBB family member (Fig. 2A, C and Supp Fig. S1). These data suggested a primary requirement of MSI2 for efficient EGFR protein expression, and indicated that endogenous MSI2 was sufficient to maximally support EGFR expression.

Activated EGFR stimulates several downstream signaling pathways, including the PI3K/AKT/mTOR/p70S6K and RAS/RAF/MEK/ERK cascades. Therefore, we analyzed the effects of MSI2 depletion on EGFR downsteam signaling in three EGFR mutant cell lines, PC9, HCC827, and H1975. MSI2 depletion reduced activity of EGFR downstream signaling in all tested cell lines (Supp Fig. S2), but the patterns of EGFR signaling suppression varied between cell models. For example, a decrease of phospho-AKT (T308) levels was observed in the H1975 and HCC827 cell lines, but not in PC9 cells, whereas a decrease of ERK and P70S6K phosphorylation was observed in the PC9 and H1975 cells, but not in HCC827 cells. In addition, we had previously demonstrated MSI2 depletion reduces expression of the TGFβ effector SMAD3 in KRAS-mutated NSCLC^9^; here, we found depletion of MSI2 reduced SMAD3 expression in all EGFR-dependent cell lines.

### MSI2 control of ERBB protein levels does not reflect alteration of ERBB mRNA levels

While the primary activity of MSI2 is as an RNA-binding protein involved in direct post-transcriptional regulation of target mRNAs^7^, it can also indirectly regulate mRNA steady state levels for some proteins by modulating the translation of transcription factors, thereby affecting transcription of their direct targets^15,16^. To discriminate between these possibilities, we examined mRNA expression of *EGFR*, *ERBB2/HER2*, and *ERBB3/HER3* following MSI2 depletion or overexpression in all of the cell line models (Supp Figs. S3 and S4). mRNA expression of EGFR individual cell lines responded to MSI2 depletion in distinct ways, including increased expression (344SQ), borderline significant decreased expression (A549 and HCC827), and no change (PC9, H1650); there was no consistent pattern across models that could explain the invariant decrease in EGFR protein and activity levels. Similar conclusions were obtained for all other conditions.

### MSI2 directly binds the EGFR mRNA

**Fig. 3:**
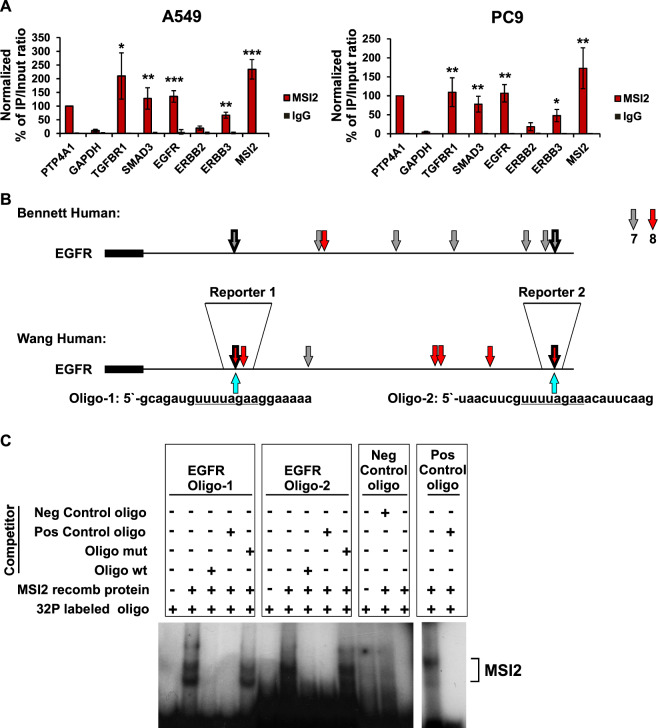
MSI2 directly binds to *EGFR* and *ERBB3* mRNA. **A** Quantification of mRNA immunoprecipitation (RIP) results from assays performed in A549 and PC9 cell lysates using antibodies to MSI2, or IgG (negative control) antibodies, followed by quantitative RT-PCR. Data are normalized to positive control *PTP4A1, TGFBR1*, and *SMAD3* are additional positive controls; *GAPDH* is a negative control. Data shown reflect the average of three independent RIP experiments. Error bars indicate SEM. Statistical analysis was performed using unpaired two tailed *t*-test. *p* < 0.05, **p < 0.01, ***p < 0.001 for all graphs. **B** Location of consensus binding sites for Musashi proteins in EGFR, as defined from studies by Bennett et al.^18^ and Wang et al.^19^. Coding sequences are represented by thick lines; 3′ untranslated regions by thin line. 7- or 8-bp consensus sequences are indicated by arrows. Thick arrows indicate identical concensus sequences identified simultaneously by Wang and Bennett studies. Shorter consensus sequences are not indicated. Blue arrows indicate the positions of ssRNA oligos (MSI2-binding sites are underscored) used for REMSA. The localization of the fragments used to generate reporter vectors are depicted as Reporter 1 and Reporter 2. **C** Analysis of recombinant MSI2 protein binding with 3′UTR fragments of EGFR mRNA by RNA-EMSA. In all, 50 ng of recombinant MSI2 protein were incubated with ^32^P-labeled ssRNA oligos, EGFR oligo 1, EGFR oligo 2, and Positive- and Negative control oligos alone, or in presence of 100-fold molar excess of unlabeled competitors, identical to the labeled probe. Competing ssRNA EGFR oligos 1 and 2 were identical to labeled probes and contained wild type (oligo wt) or mutant (oligo mut) MSI2-binding motifs.

These data suggested that the main biologic role of MSI2 would be direct translational regulation for the EGFR mRNA. To test this hypothesis, we performed RNA immunoprecipitation assays (RIP) with an MSI2 antibody coupled with qRT-PCR in two cell lines, A549 and PC9 (Fig. 3A), using three previously defined MSI2 target mRNAs (*PTP4A1*, *SMAD3*, and *TGFβR1*^9,17^) as positive controls, and *GAPDH* as a negative control. Antibodies to MSI2 specifically immunoprecipitated the *EGFR* mRNA as efficiently as they did the positive controls (Fig. 3A). MSI2 antibody also immunoprecipitated the *ERBB3* mRNA, although to a lesser degree than EGFR, and did not significantly immunoprecipitate the ERBB2 mRNA. Interestingly, MSI2 also robustly immunoprecipitated its own transcript (Fig. 3A).

Consensus sequences for MSI2 binding have been proposed by Bennett et al.^18^, and Wang et al.^19^, based on high-throughput sequencing of *RNA* isolated by crosslinking immunoprecipitation (HIT-CLIP) profiling of target MSI2 mRNAs in mouse cell lines, and described as partially degenerate motifs of 3 to more than 8 nucleotides (Supp Tables S1 and S2). We performed in silico analysis of the EGFR, ERBB2, and ERBB3 mRNAs versus the positive and negative controls, searching for occurrence of the longer (7 or 8 nucleotides) binding consensus sites, as these were less likely to occur by chance (Fig. 3B and Supp Fig. S5). Among the human ERBB family mRNAs, the EGFR mRNA had multiple copies of the MSI2-binding consensus, rivaling the positive controls; ERBB3 had a limited number of candidate binding sites, while ERBB2 had only one, paralleling the results observed from RNA-IP (RIP). Similar results were found through in silico analysis of the mRNA encoding the murine Egfr, Erbb2, and Erbb3 genes (Supp Tables S1 and S2).

Next, we analyzed the binding of MSI2 recombinant protein with 3′UTR fragments of EGFR mRNA by RNA-EMSA. We have defined two mRNA regions harboring 8-bp MSI2-binding motifs which sequences were identified simultaneously by Wang and Bennett studies^18,19^ (Fig. 3B). Therefore, for RNA-EMSA we used two independent ssRNA oligonucleotides (oligos) derived from EGFR consensus sequences and their mutated analogs corresponding to those regions (Supp Table S3). Recombinant MSI2 protein bound to both EGFR-derived oligos (Fig. 3C). We also observed MSI2 binding to a positive control RNA oligo comprised of a 6x repeat of MSI2 core binding site, UAG, but not to a negative control RNA oligo corresponding to 3′UTR fragment of VEGFR2 mRNA lacking any variants of MSI2-binding motifs (Fig. 3C). These data support the idea that MSI2 directly binds to the *EGFR* mRNA. Subsequent assessment whether fusion of short 3′ sequences from EGFR containing individual MSI2-binding sites was sufficient to influence expression of a luciferase reporter gene yielded negative results (Supp Fig. S6), suggesting combination effect of multiple sites may be important for functional control of translation, as has been reported for other MSI2 targets^20^.

### MSI2 depletion selectively reduces the growth of NSCLC cells with activating mutations in EGFR

**Fig. 4:**
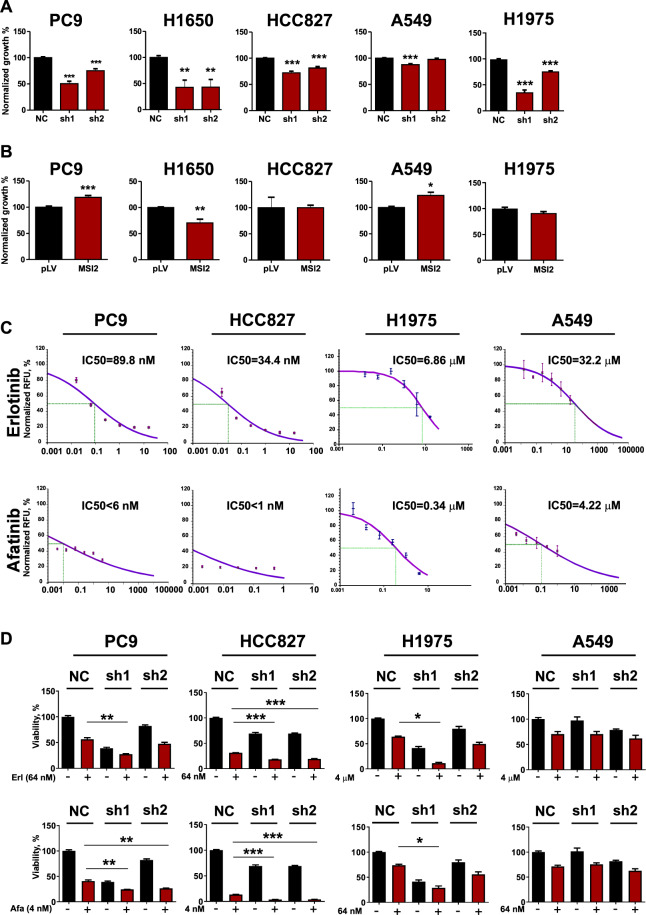
MSI2 supports proliferation and resistance to drugs inhibiting EGFR in EGFRmut NSCLC cell lines. **A** Cell viability quantified by Cell Titer Blue (CTB) assay, in indicated cell lines with negative control (NC) for depletion, or depletion of MSI2 (sh1, sh2). **B** Quantification of viability by CTB assay, 96 h after doxycycline treatment, in cell lines expressing empty lentivirus (pLV) or the same vector overexpressing MSI2. **C** IC50 curves for viability of cell lines measured by CTB assay following 96 h treatment with erlotinib or afatinib. Representative data of one of three independent experiments are presented. **D** EGFR^mut^ (PC9, HCC827, and H1975) and KRAS^mut^ (A549) cell line derivatives expressing doxycycline-inducible anti-MSI2 shRNAs (sh1 and sh2) or negative control (NC) cells were incubated in complete medium in presence of 1 μg/ml of Doxycycline with indicated concentrations of erlotinib (Erl) or afatinib (Afa) for 96 h, then viability measured by CTB Assay. For **A**, **B**, and **D**, data presented represent the average of three independent experiments. Error bars represented by SEM. Statistical analysis was performed using unpaired two tailed *t*-test. *p < 0.05, **p < 0.01, ***p < 0.001 for all graphs.

We previously reported that in 3 NSCLC cell lines dependent on activating mutation of *KRAS* (344SQ, A549, and H358), depletion of MSI2 had minimal or no effect on cell proliferation in vitro^9^. Given EGFR^mut^ NSCLC cell lines are strongly dependent on activity of EGFR^14^, we hypothesized that reduced MSI2 expression may reduce viability in these models. We examined the effects of MSI2 depletion on proliferation of the EGFR^mut^ PC9, HCC827 and H1650 human NSCLC cell lines, each of which is highly sensitive to EGFR tyrosine kinase inhibitors (TKIs)^21^. Proliferation of these cell lines was suppressed by MSI2 depletion with doxycycline-inducible shRNAs (1.5–2.5 fold in all models; *p* < 0.0001 for PC9, H1975, and HCC827, and *p* < 0.0096 for H1650), whereas no significant effect was observed in *RAS*-mutated A549 cells (Fig. 4A). In contrast, overexpression of MSI2 did not consistently affect cell proliferation in any cell model (Fig. 4B). These viability effects were not related to variation in the intrinsic expression level of either MSI2 or EGFR in the assessed cell lines (Supp Fig. 7).

### MSI2 inhibition specifically sensitizes EGFR^mut^ NSCLC cell lines to anti-EGFR TKIs in vitro

As EGFR^mut^ NSCLC is uniquely sensitive to EGFR-targeting inhibitors, we hypothesized that targeting MSI2 might influence the efficacy of small molecule EGFR tyrosine kinase inhibitors in these cells. Erlotinib is a reversible and afatanib an irreversible inhibitor of EGFR tyrosine kinase^3^. We performed initial IC_50_ determinations for erlotinib and afatinib in PC9, H1975, and HCC827 and a contrasting insensitive KRAS-mutated line, A549 (Fig. 4C). At clinically achievable doses of drug selected to be close to the IC_50_ values in the EGFR^mut^ cell lines, MSI2 depletion significantly lowered cell viability of cells treated with erlotinib or afatinib after 3 days in the presence of drugs (Fig. 4D). In contrast, neither administration of EGFR inhibitor, MSI2 depletion, or the combination significantly affected the growth of A549 cells (Fig. 4D).

To analyze the longer-term consequences of MSI2 depletion, we performed clonogenic cell survival assays in cells with or without stable MSI2 depletion, with or without drug treatment (Supp Fig. S8), using PC9 and A549 cells. In the EGFR^mut^ PC9 cells, treatment with either erlotinib or afatinib significantly reduced growth, as did depletion of MSI2. Notably, drug treatment and MSI2 depletion in combination more significantly reduced clonogenic viability than either treatment alone. In contrast, neither drug treatment or MSI2 depletion significantly affected clonogenic capacity in KRAS-mutated A549 cells (Supp Fig. S8). To additionally probe the effect of MSI2 depletion on cellular response to EGFR inhibition, we also analyzed cells treated with the third generation anti-EGFR TKI, osimertinib, which is specifically active against cells bearing a T790M mutation in EGFR, and less active in cells with wild-type EGFR^22^. The IC50 value of osimertinib was established as 5.3 nM in PC9 cells, and 3.3 nM in H1975 cells (Supp Fig. S9). At doses of drug selected to be close to the IC_50_, MSI2 depletion significantly lowered term viability in PC9 cells treated with osimertinib, while a much more limited effect on viability was observed in H1975 cells (Suppl Fig. S9).

### Xenograft analysis of MSI2 inhibition indicates in vivo combination effect with EGFR-targeting drugs in EGFR^mut^ NSCLC cells

**Fig. 5:**
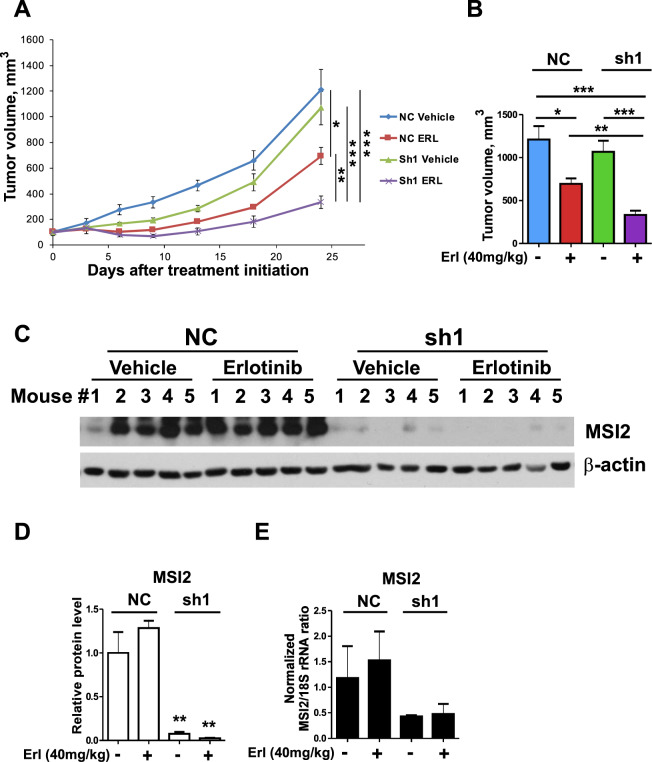
MSI2 knockdown increases the sensitivity of EGFR mutant xenograft tumors to erlotinib treatment. **A** Growth curve of subcutaneous xenografts PC9 cells stably expressing lentiviral vector as negative control (NC) or shRNA to MSI2 (sh1), and treated with vehicle or erlotinib (ERL) for 24 days. *N* = 5/group. **B** Quantification of tumors at endpoint of experiment in **A**. **C** Western blot analysis of MSI2, protein levels from treated tumors. **D** Quantification of western blot data from **C**; data normalized to β-actin. **E** Quantitative RT-PCR of mRNA collected from indicated xenograft tumors at the end of experiments. Negative controls are denoted NC. Data are normalized to 18S rRNA, as noted. Relative quantification (RQ) of gene expression was performed using 2^−ΔΔCt^ method. Data are presented as normalized average RQ means in each group (*n* = 5) of animals. In all graphs, error bars represented by SEM. Statistical analysis was performed using unpaired two tailed *t* test. *p < 0.05, **p < 0.01, ***p < 0.001 for all graphs.

To determine whether the combination effect of MSI2 depletion and EGFR inhibitors on EGFR^mut^ cell proliferation also occurs in vivo, we performed xenograft analyses using EGFR^mut^ PC9 cells derivatives with doxycycline-inducible shRNA-dependent MSI2 depletion, or matched empty vector. When tumors reached 150 mm^3^, mice were dosed for 21 days with either erlotinib (40 mg/kg, 5 days a week) or vehicle. Consistent with our in vitro observations, the depletion of MSI2 in this model significantly reduced tumor growth, and significantly enhanced the anti-tumor effect of erlotinib (Fig. 5A, B). Western analysis confirmed efficient depletion of MSI2 through the duration of the experiment (Fig. 5C–E). In contrast, in similar experiments performed in the KRAS-dependent A549 cell line, neither MSI2 depletion, erlotinib treatment, nor the combination significantly impaired tumor growth (Supp Fig. S10A, B). MSI2 depletion was sustained through the experimental endpoint (Supp Fig. S10C–E).

### Correlated expression of MSI2 and EGFR in human EGFR^mut^ NSCLC

**Fig. 6:**
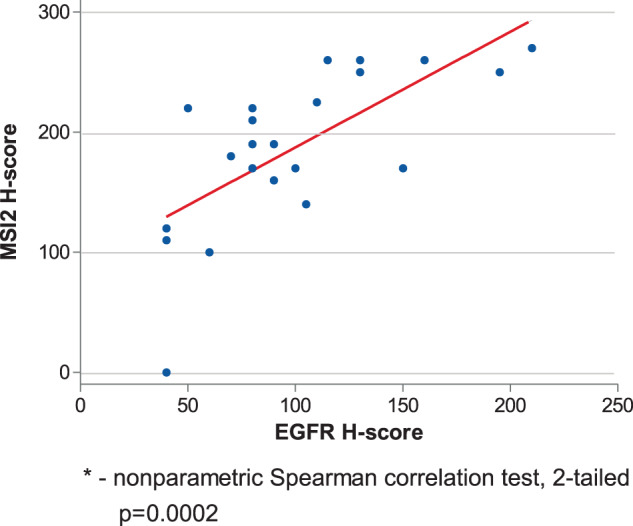
Expression of MSI2 and EGFR proteins in human NSCLC primary tumors. **A** H scores for MSI2 and EGFR in EGFR^mut^ NSCLC tumor TMA samples (see Supp Table S4 for clinical characteristics). For MSI2 and EGFR IHC quantification, each spot was examined by board-certified pathologists (ED and NK) who assigned a score of 0 (no staining), 1+ (weak staining), 2+ (moderate staining), and 3+ (strong staining) within carcinomatous areas. The score for each of the two tumor spots was averaged for statistical analysis. The H-score, which ranges from 0 to 300, was calculated using the following formula: [1(% cells 1+) + 2 (% cells 2+) + 3 (% cells 3+)], which reflects staining intensity as well as percentage of positive cells^46,47^.

To specifically evaluate the relationship between MSI2 and EGFR correlation in the EGFR^mut^ subset of NSCLC tumors, we performed IHC studies of EGFR and MSI2 expression in an independent group of 22 EGFR^mut^ NSCLC tumors (Fig. 6 and Supp Table S4). Here, in spite of the small size of this cohort, Spearman’s analysis of H-scores indicated a significant positive correlation between MSI2 and EGFR expression levels: Spearman rank: 0.7158 (*p* = 0.0001799).

## Discussion

Despite significant progress in therapeutic management of EGFR^mut^ NSCLC, the long term (>5 years) survival of patients with advanced EGFR^mut^ NSCLC remains low (<10%)^23^. Therapeutic options for EGFR^mut^ NSCLC patients remain limited, with otherwise promising immunotherapies having low response rates in this population due to lower levels of PD-L1 and lower tumor mutational burden^24^. Improving outcomes in this population is of great importance and represents an unmet need. Agents targeting EGFR in EGFR^mut^ tumors are one of the few effective targeted therapies for use as a frontline therapy. In the current study, we for the first time identify MSI2 as a regulator of EGFR expression. This interaction is based on a mechanism by which MSI2 binds directly to consensus motifs within the *EGFR* mRNA and promotes EGFR translation. As a result, the proliferation of EGFR^mut^ cells depends on expression of MSI2 in vitro and in vivo, and MSI2 depletion enhances the activity of EGFR-targeted inhibitors in EGFR^mut^ NSCLC. Overall, our work suggests that blockade of MSI2 could be of specific therapeutic value in EGFR^mut^ tumors.

While initially highly effective in appropriately selected NSCLC populations, durable long term responses to first generation EGFR inhibitors such as erlotinib are relatively uncommon, and most patients invariably progress to drug-resistant disease, typically within ~12 months^3,25,26^. The predominant mechanism of acquired resistance to these inhibitors, accounting for ~50% of all cases, is the acquisition of a secondary mutation (T790M) in exon 20 of EGFR gene. Osimertinib is a third generation, highly potent EGFR inhibitor, active against common EGFR mutations, and also the T790M EGFR mutation, which was initially FDA-approved in 2d line for EGFR^mut^ patients with T790M mutations and in 2018 received approval as a first line therapy for EGFR^mut^ NSCLC^4^. While this new agent significantly prolongs the survival of this patient group^4^, resistance almost inevitably emerges through a variety of alternative mechanisms including amplification of *EGFR*, *MET*, and *KRAS*, as well as mutations activating EGFR effectors including MEK1, KRAS, JAK2, PIK3CA, and other proteins^27,28^. In this context, identifying ways to inhibit or downregulate EGFR and potentially some of its resistance-conferring effectors may be a productive approach.

In concept, one way to downregulate EGFR is to reduce its transcription. The *EGFR* promoter is regulated by transcription factors including SP1, AP-2/TFAP2A, TP53, WT1, IRF1, and others^29^; however, these proteins have proven difficult to target. Another way to downregulate EGFR therapeutically would be to use a proteolysis targeted chimera (PROTAC) approach, leading to targeted degradation of the protein through induced ubiquitin-mediated destruction^30^. While this approach is promising, it solely targets EGFR, leaving open the possible of resistance associated with activation or upregulation of EGFR effectors, or other RTKs such as MET that can compensate for loss of EGFR. Our results demonstrate that inhibition of MSI2 is particularly promising as an alternative approach, as MSI2 both sustains EGFR translation, and also is important for translation of other proteins which typically support resistance to EGFR-targeting TKIs, including MET, MEK, mTOR, and others. Hence, the pleiotropic effects of MSI2 inhibition could limit the capacity of some of these convergent and/or parallel signaling pathways to overcome EGFR resistance. This complex activity may explain why MSI2 inhibition is particularly important for viability of EGFR-dependent lines in the experiments reported here. MSI2 depletion consistently reduced EGFR expression. In analysis of downstream EGFR effectors, the effect of MSI2 depletion was heterogeneous, with the PI3K and RAF/MEK/ERK signaling cascades variably inhibited amongst the different cell lines, likely due to variation in the genetic and epigenetic context of the various models. However, MSI2 depletion resulted in SMAD3 depletion in all cell lines (Supp Fig. 2), and SMAD3-dependent signaling has been reported to collaborate with signaling by EGFR direct effectors to sustain viability of EGFR-dependent cells^31^. Hence, MSI2 loss may cause loss of viability of EGFR-dependent cells by targeting multiple factors contributing to survival.

It is also of interest that high expression of EGFR^mut^ correlated with elevated MSI2 expression in human NSCLC, which supports a likely role of MSI2 in maximizing EGFR translation under conditions in which the protein serves as oncogenic driver. Surprisingly, in spite of intense study of EGFR and other ERBB proteins, relatively little is known about the regulation of EGFR protein translation. One study has shown that hypoxia/HIF2α-induced translation of EGFR mRNA represents a common mechanism for EGFR overexpression in solid tumors^32^. A later study identified an RNA hypoxia response element (rHRE) in the 3′ end of EGFR mRNA; binding of a complex containing HIF2α, the RBP RBM4, and the cap-binding protein eIF4E2 to this motif and targets the mRNA to polysomes for active translation,thereby evading hypoxia-induced repression of protein synthesis^33^. Distinct from regulation by hypoxia, active phospholipase D2 (PLD2) has been shown to increase expression of EGFR in in breast cancer, in part via stabilization of its mRNA, although a direct mechanism was not identified^34^. To this limited set of EGFR translational regulators, our study adds MSI2. No prior studies of MSI2-bound transcripts have identified EGFR^18,19^, and the precise mechanism by which MSI2 influences mRNA translation of EGFR will require further study. In work on other Musashi targets, MSI2 and its homolog MSI1 can function as a translational activator or repressor, due to the context of binding motifs for additional cofactors, and the abundance of those cofactors in distinct cell types, with most of the work addressing MSI1^6,10,11^. Further work will be needed to understand the molecular mechanism governing MSI2 enhancement of EGFR translation.

In an additional finding of interest, RIP analysis indicates that MSI2 binds to its own mRNA, raising the interesting possibility that it regulates its own translation. The phenomenon of autoregulation, or autogenous regulation, has been described as of 2014 for at least 57 RBPs, and has been associated with a number of distinct post-transcriptional mechanisms which usually lead to inhibition of translation^35^. The specific mechanisms potentially involved in MSI2 autoregulation and their relevance to NSCLC require further investigation. Efforts to target MSI2 with small molecule agents are in progress^7,36,37^, and based on this study, these agents would be of particular importance in EGFR^mut^ NSCLC. Our in vitro data support the potency of MSI2/EGFR inhibitor combinations. Certainly, given the promising results of this study, our findings justify further exploration of MSI2 signaling mechanism and development of strategies of targeting MSI2 for use in EGFR^mut^ NSCLC, and potentially other tumor types.

## Matherials and methods

### RPPA

The 344SQ-SCR, 344SQ-m1, and 344SQ-m2 and 393P-SCR, 393P-m2a, and 393p-m2b mouse cells were previously described^9^. Prior to analysis, these cells were lysed and prepared according to MD Anderson Core Facility instructions^38–40^, and RPPA was performed at MD Anderson facility and previously published^9^. Data were visualized using the MultiExperiment Viewer program (www.tm4.org/mev.html)^41^.

### Vector construction and lentivirus production

To generate stable cell lines with inducible MSI2 knockdowns, self-complementary single-stranded DNA oligos (Supp Table S5) were annealed and cloned into AgeI/EcoR1 sites of Tet-pLKO-puro vector (Addgene plasmid # 21915). MSI2 ORF (NM_138962.2) was amplified by PCR with specific primers and high-fidelity Ex Taq DNA polymerase (Takara Bio USA, Inc., Mountain View, CA) using a cDNA containing human MSI2 obtained from OriGene (Rockville, MD) as a template, and cloned into XbaI/XhoI sites of pLV-CMV-puro vector (a kind gift from Dr. A. Ivanov). All generated cell lines used in the study are noted in Supp Table S6. All constructs were validated by direct sequencing.

### SiRNA transfections

SiRNAs targeting human MSI2 (Supp Table S7) and nonspecific control pool siRNAs were purchased from Qiagen (Frederick, MD). Cultured cells at 50% confluence were transfected with siRNA at final concentrations of 50 nmol/L using the Lipofectamine RNAiMAX transfection reagent (Thermo Fisher Scientific, Waltham, MA) according to the manufacturer’s instructions.

### Cell culture

Murine NSCLC cell lines (344SQ and 393p) from *Trp53*^*R172HΔG/+*^*Kras*^*LA1/+*^mice were previously described^9^. Human alveolar basal epithelial adenocarcinoma cell lines including a KRAS-mutated cell line (A549), three cell lines bearing EGFR exon 19 deletions (PC9, HCC827, and H1650), and an EGFR-mutated line with an activating L858R mutation in exon 21 and T790M mutation in exon 20 (H1975) were obtained from the American Type Culture Collection (ATCC). Initial stocks were cryopreserved, and at every 6-month interval a fresh aliquot of frozen cells was used for the experiments. No additional authentification was performed. We note, for PC9 and HCC827, EGFR was amplified as well as mutated^42,43^. All cells were cultured in RPMI 1640 (Bio-Whittaker, Walkersville, MD) supplemented with 10% FBS (Hyclone, Logan, UT), penicillin (100 U/ml), streptomycin (100 μg/ml), sodium pyruvate (1 mM), and non-essential amino acids (0.1 mM) under conditions indicated in the figure legends.

Prior to Western analysis, for MSI2 overexpression, control (pLV/pLD) and paired MSI2-overexpressing NSCLC cell lines were incubated in complete medium for 24 h. For MSI2 depletion by shRNA, stable negative control (NC) cell lines or those expressing doxycycline-inducible shRNAs (sh1 and sh2) targeting MSI2 were cultured in complete medium in the presence of 1 μg/ml of doxycycline for 48 h. In H1650 cells, MSI2 depletion was in some cases accomplished by siRNA, and MSI2 overexpression by direct lentiviral infection without clonal selection, because of lethality associated with sustained manipulation of MSI2. For depletion, H1650 cells were transfected with negative control siRNA oligos (GL2) or with pooled anti-MSI2 siRNA oligos (h1 and h2) followed by incubation in complete medium for the next 48 h. For MSI2 overexpression, H1650 cells were transfected with an empty vector (pLV) or with a vector encoding the MSI2 open reading frame, and incubated in complete medium for 24 h.

### Antibodies and drugs

Anti-MSI2 (#ab76148), anti-MSI1 (#ab21628), and anti-β-actin HRP conjugated (#ab49900) antibodies and recombinant MSI2 protein (#ab167853) were obtained from Abcam, (Cambridge, UK). Anti-EGF receptor (#4267), phospho-EGFR (Y1068) (mAb #2234), HER3/ErbB3 (#12708), HER2/ErbB2 (#4290), Smad3 (#9523), phospho-AKT (T308) (#13038), total AKT (#2920), phospho-ERK (T202/Y204) (#4370), total ERK (#4696), phospho-p70S6K (T389) (#9234), total p70S6K (#2708), and normal Rabbit IgG (#2729) were obtained from Cell Signaling, (Danvers, MA). Erlotinib and afatinib were obtained from LC Laboratories (Woburn, MA), doxycycline from Sigma-Aldrich (D9891, Darmstadt, Germany). SUPERase-In RNAse inhibitor was obtained from Thermo Fisher Scientific, (AM2694 Waltham, MA). Osimertinib was obtained from Selleckchem (#S7297, Houston, TX)

### mRNA expression

Total DNA-free RNA was isolated using Quick-RNA™ MiniPrep (#R1054) (Zymo Research, Orange, CA), reverse transcribed using Moloney murine leukemia virus reverse transcriptase (Ambion-Thermo Fisher Scientific, Waltham, MA) and a mixture of anchored oligo-dT and random decamers (Integrated DNA Technologies, Coralville, IA). Gene expression was analyzed by quantitative RT-PCR, using the primers listed in Supp Table S8.

### Western blots

Cell lysates preparation and western Blot analysis were performed using standard methods as previously described^9^. Image analysis was done using ImageJ (National Institutes of Health, Bethesda, MD), with signal intensity normalized to β-actin or total level of detected proteins. Data were analyzed in Excel and GraphPad Prism by unpaired *t*-test to determine statistical significance.

### Cell proliferation and viability assays

To analyze the effects of MSI2 depletion or overexpression on proliferation of NSCLC cells, cells (500–1000 cells/well) were plated in 96-well cell culture plates in complete media. After 24 h, the expression of specific shRNAs was induced by addition of 1 μg/ml of Doxycycline, and a CellTiter-Blue® assay (Promega, Fitchburg, WI) to obtain a 0 h time point. Final measurements were performed at a 96 h time point, and normalized to 0 h data. To analyze the effect of erlotinib and afatinib on viability of MSI2-depleted NSCLC cells, experiments were performed with or without addition of drug at the 0 h time point. For the PC9 and HCC827 cell lines, we used drug concentrations corresponding to the average of IC50 values determined for those cell lines. For the H1975 cell line we used drug concentrations corresponding to IC50 values determined for this cell line. All assays were performed in three technical repeats, and in three biological repeats.

### Clonogenic survival assays

PC9 and A549 cells (200 cells per well) were plated in 12-well plates and incubated in complete media. After 24 h the expression of MSI2-targeting shRNAs was induced by addition of 1 μg/ml of doxycycline, and cells were incubated for 7–14 days. Cells were fixed in 10% acetic acid/10% methanol solution and stained with 0.5% (w/v) crystal violet as previously described^44^. A colony was defined as consisting of >50 cells, and counted digitally using ImageJ software as described previously^45^.

### RNA-IP assays and quantitative PCR

RNA was immunoprecipitated from cell lysates (2 × 10^7^ cells per IP) using either a control normal rabbit IgG or rabbit monoclonal anti-MSI2 antibody and the Magna RIP RNA-binding Protein Immunoprecipitation kit (cat#17-700, Millipore, Burlington, MA). Manufacturer’s instructions were followed with the exception that RNeasy MinElute Cleanup kit (cat#74202, Qiagen, Venlo, Netherlands) was used to prepare RNA. Immunoprecipitated RNAs were quantified by quantitative PCR (qPCR) using primers indicated in Supp Table S8, using PTP4A1as a normalization (positive) controland GAPDH as a negative control.

### In silico evaluation of MSI2 binding to EGFR mRNA

Human and murine genome sequences for *EGFR* were obtained from the UCSC Human Gene Sorter December 2013 (GRCh38/hg38) assembly, and scanned for Musashi binding motifs previously defined by Bennett et al.^18^ (15 motifs with highest *p* values) and Wang et al.^19^ (8 motifs with highest *p* values; Supp Tables S1 and S2).

### Analysis of recombinant MSI2 protein binding: RNA-EMSA and luciferaase assays

One microgram of each ssRNA oligos (Suppl Table S8) was labeled with 20 μCi of [γ-^32^P]ATP using T4 Polynucleotide kinase (New England Biolabs, Ipswich MA) and purified on a Sephadex G25 MicroSpin column (GE Healthcare Ltd, Buckinghamshire UK). In all, 50 ng of recombinant MSI2 protein were equilibrated for 10 min at 25 °C in 20 μl of binding buffer containing 10 mM HEPES (pH-7.6), 20 mM KCl, 1 mM MgCl_2_, 1 mM DTT, 5% Glycerol, and 1× Protease Inhibitors Cocktail with 2 μg of tRNA and 20 units of SUPERase-In RNAse inhibitor. Twenty nanograms (10–20,000 Cpm) of ^32^P-labeled ssRNA oligos corresponding to 3′UTR of EGFR mRNA regions harboring 8-bp MSI2-binding motifs added and incubated for another 20 min at 25 °C. In competition assays 50 ng of recombinant MSI2 protein were pre-incubated with binding buffer and 100-fold molar excess of unlabeled ssRNA oligos identical to the labeled probe containing wild-type or mutant MSI2-binding motifs. Reaction mixtures were separated using a 8% polyacrylamide gel in 0.5×TAE. Separated complexes were detected by autoradiography after the gels were dried onto filter paper.

To assess functionality of individual MSI2-binding sites, we used a Dual Glo Luciferase assay (Promega, Madison, WI). We have generated four reporter vectors based on pGL3-promoter vector backbone by inserting two (250 bp and 318 bp) EGFR 3′UTR fragment spanning MSI2 consensus motifs corresponding to oligo #1 and #2 and their mutant analogs downstream of firefly luciferase. The localization of two fragments is depicted in the Fig. 3B. Assays were performed according to manufacturer’s instructions, with Firefly luciferase expression normalized to that of a Renilla luciferase control.

### Assessment of in vivo tumor growth

For in vivo studies, 4 × 10^6^ of PC9 or A549 cells stably transfected with pLV vector only, or pLV-MSI2 shRNA, were injected subcutaneously (s.c.) in the flank region of 6-week-old male C.B17/Icr-scid mice using a 27-gauge needle. All animal procedures were done in accordance with institutional guidelines on animal care and with appropriate institutional certification. Animals were fed sterile AIN-93M diet (Harlan Teklad, Madison, WI) and water ad libitum. When tumor volumes reached ~150 mm^3^, animals were randomly assigned to the control or experimental groups (*n* = 5 mice/group). The mice were treated with (i) 0.15 M NaCl with 10% (2-Hydroxypropyl)-β-cyclodextrin (HPCD) solution (vehicle); or (ii) erlotinib (40 mg/kg in 0.15 M NaCl with 10% HPCD solution, daily, by oral gavage); in addition, all mice were treated with 20 mg/kg of doxycycline in water (daily, P.O.). Tumors were measured twice weekly and their volumes were calculated with the formula: [volume = 0.52 × (width)^2^ × length]. No blinding was done. After 24 days, mice were euthanized and tissues were collected for analysis.

### Immunohistochemistry of human NSCLC

For analysis of patients EGFR^mut^ NSCLC specimen, EGFR mutations were confirmed by PCR using a Therascreen EGFR RGQ PCR Kit (Qiagen, Frederick, MD), and surgical specimens from the Rostov Research Institute Human Tissue Repository Facility (HTRF) were used. At the time of tissue acquisition, patients provided Institutional Review Board (IRB)–approved informed consent for storing tissue and reviewing deidentified clinical data. Clinical information (Supp Table S4) from the repository database was abstracted in an anonymized fashion.

Tissue samples were stained for EGFR and MSI2 via immunohistochemical (IHC) approach and and hematoxylin and eosin (H&E) stained sections were used for morphological evaluation purposes, and unstained sections were used for IHC staining using standard methods. Briefly, 5 µm formalin-fixed, paraffin-embedded sections were deparaffinized and hydrated. Sections were then subjected to heat-induced epitope retrieval with 0.01 M citrate buffer (pH 6.0) (MSI2) or EDTA buffer (EGFR). Endogenous peroxidases were quenched by the immersion of the slides in 3% H_2_O_2_ solution. The sections were incubated overnight with primary antibodies to MSI2 (EP1305Y, Rabbit, 1:100, Abcam #ab76148), EGFR(D38B1, Rabbit, 1:50, Cell signaling, Cat #4267) at 4 °C in a humidified slide chamber. As a negative control, the primary antibody was replaced with normal mouse/rabbit IgG to confirm absence of specific staining. Immonodetection was performed using the Dako Envision+ polymer system and immunostaining was visualized with the chromogen 3, 3′-diaminobenzidine. All slides were viewed with a Nikon Eclipse 50i microscope and photomicrographs were taken with an attached Nikon DS-Fi1 camera (Melville, NY, USA).

### Statistical analysis

Integration of clonogenic survival as a function of dose, or area under the curve, was calculated using GraphPad Prism Software, as were assessments of protein or mRNA expression. We used Spearman correlation analysis for Fig. 6.

## Supplementary information

Supplementary figures
