# Supplementary material for: Musashi-2 (MSI2) regulates epidermal growth factor receptor (EGFR) expression and response to EGFR inhibitors in EGFR-mutated non-small cell lung cancer (NSCLC)
Source: Oncogenesis. 2021 Mar 15;10(3):29. doi: 10.1038/s41389-021-00317-y (PMC7961039; doi:10.1038/s41389-021-00317-y)
Supplement: Supplementary file 1 — Supplementary figures [file 41389_2021_317_MOESM1_ESM.pdf]

# Makhov et al. Musashi-2 (MSI2) regulates epidermal growth factor receptor (EGFR) expression and response to EGFR inhibitors in EGFR-mutated non-small cell lung cancer (NSCLC).

**Supplementary Figures and Tables.**

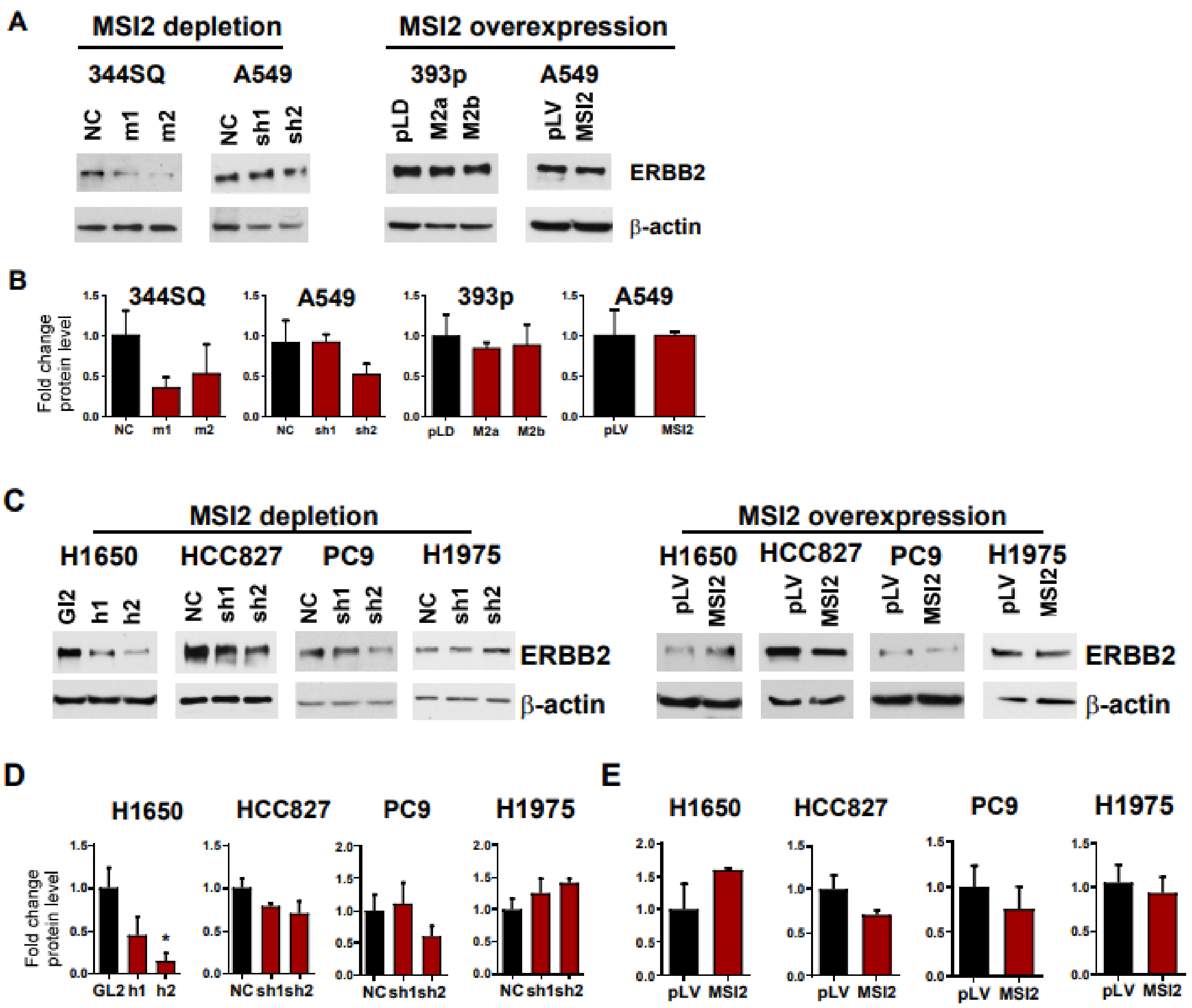


**Supplementary Figure S1. MSI2 regulation of ERBB2/HER2 protein expression.** Representative western blot (**A, C**) and quantitation (**B, D**) for ERBB2 expression in indicated cell lines, following depletion (m1, m2, sh1, sh2, h1, h2) or overexpression (M2a, M2b, MSI2) of MSI2. NC and GL2 are negative controls for MSI2 depletion; pLenti63/V5 DEST (pLD) and pLV-CMV-puro (pLV) are negative controls for MSI2 overexpression. Normalized quantification of Western blot data is from at least three independent experiments by Image J software. Error bars represented by SEM. Statistical analysis was performed using unpaired two tailed t-test. *, p<0.05, **, p<0.01, ***, p<0.001 for all graphs.

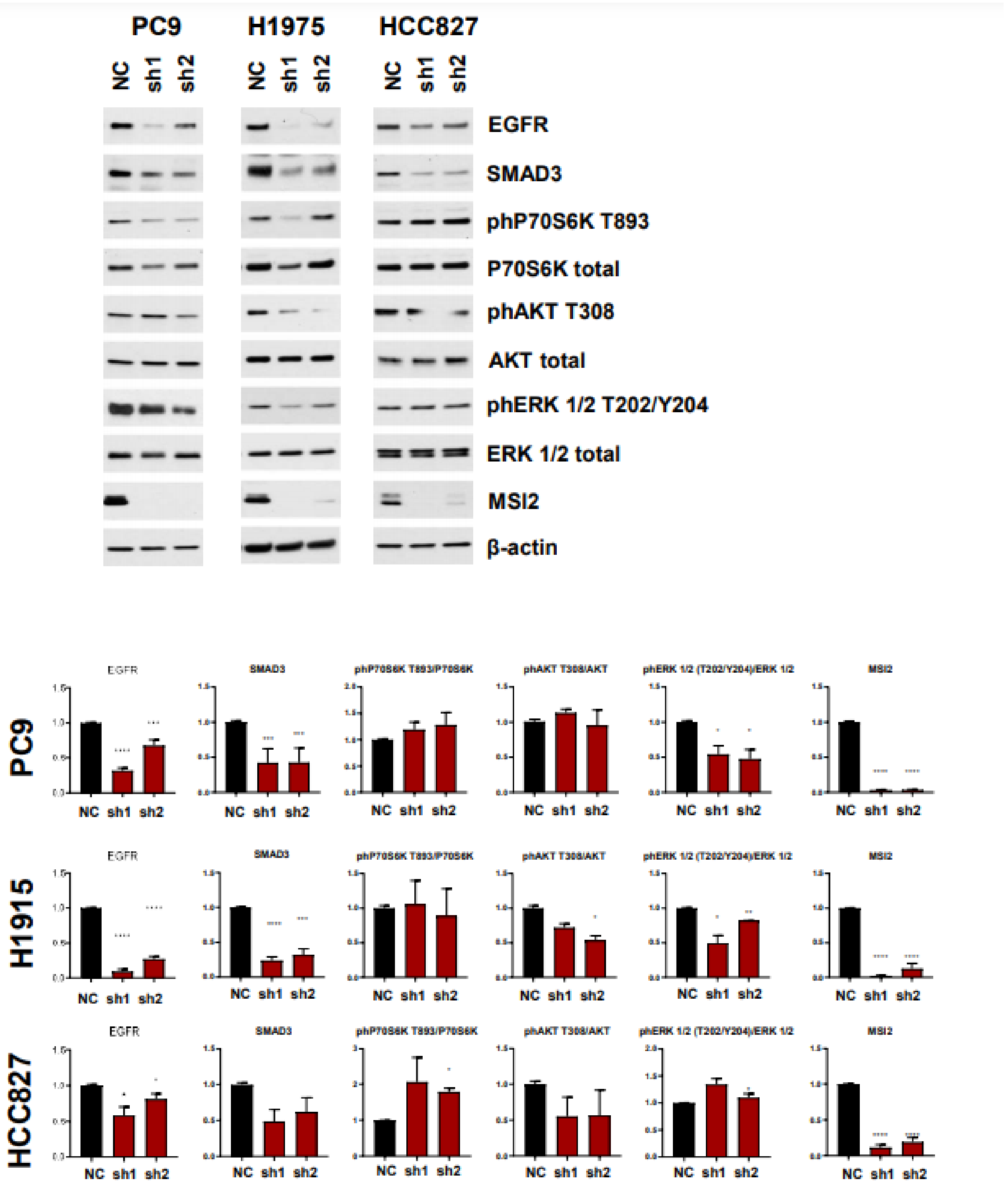


**Supplementary Figure S2. Consequences of MSI2 depletion on EGFR downstream signaling in EGFR mutant NSCLC cell lines.** Representative western blot (A) and quantitation (B) for phospho-/total levels of EGFR downstream effectors and SMAD3 expression in indicated cell lines, following MSI2 depletion (sh1, sh2). NC is negative control for MSI2 depletion. Normalized quantification of Western blot data is from at least three independent experiments by Image J software. Error bars represented by SEM. Statistical analysis was performed using unpaired two tailed t-test. *, $p<0.05$, **, $p<0.01$, ***, $p<0.001$ for all graphs.

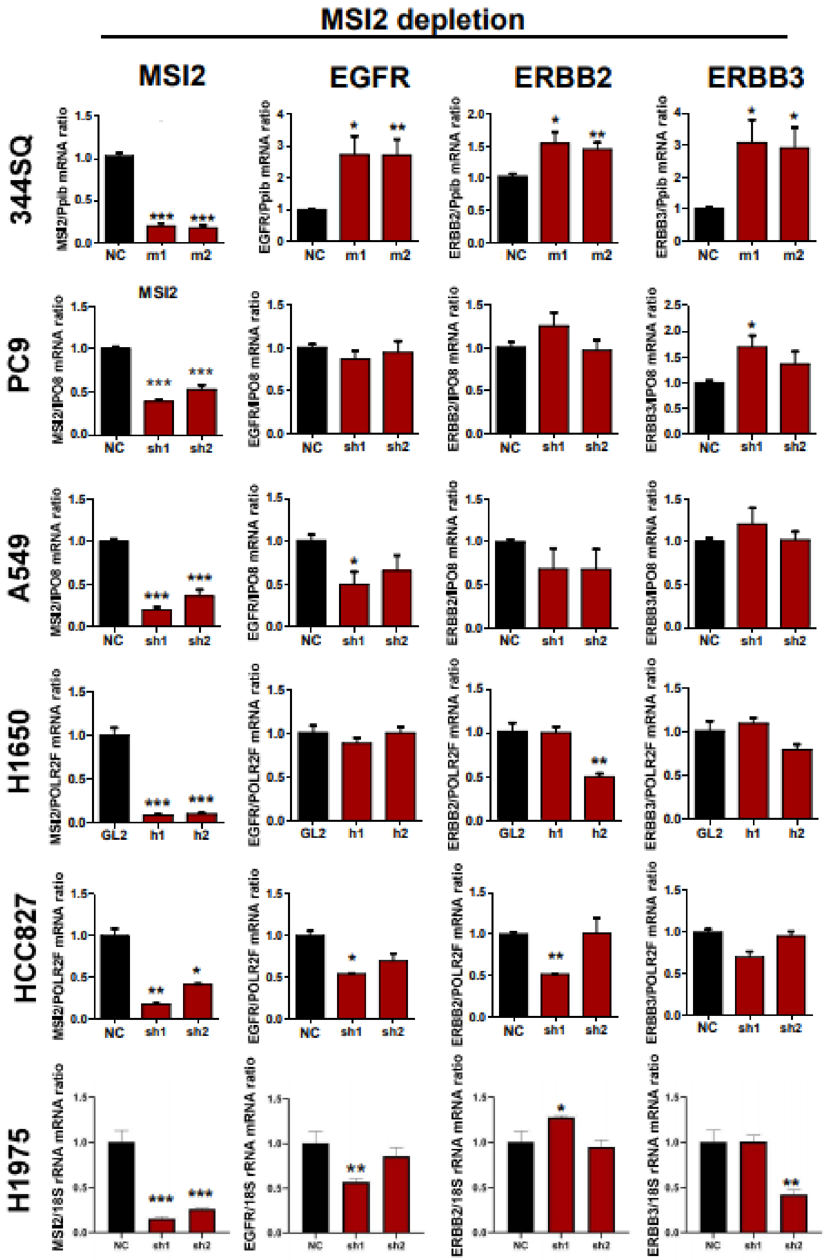


**Supplementary Figure S3. Consequences of MSI2 depletion on mRNA expression of ERBB genes in NSCLC cell line models.** Quantitative RT-PCR of mRNA collected from indicated cell lines, following MSI2 depletion by shRNA (m1, m2, sh1, sh2) or siRNA (h1, h2) in two KRAS$^{mut}$ (344SQ and A549) and three EGFR$^{mut}$ (H1650, HCC827, and PC9) NSCLC cell lines. Negative controls are denoted GL2 and NC. Data are normalized to Ppib, POLR2F, or IPO8, as noted. Relative quantification (RQ) of gene expression was performed using $2^{-\Delta\Delta Ct}$ method. Data are presented as average RQ means of three independent qRT-PCR experiments. Error bars represented by SEM. Statistical analysis was performed using unpaired two tailed t-test. *, $p<0.05$, **, $p<0.01$, ***, $p<0.001$ for all graphs.

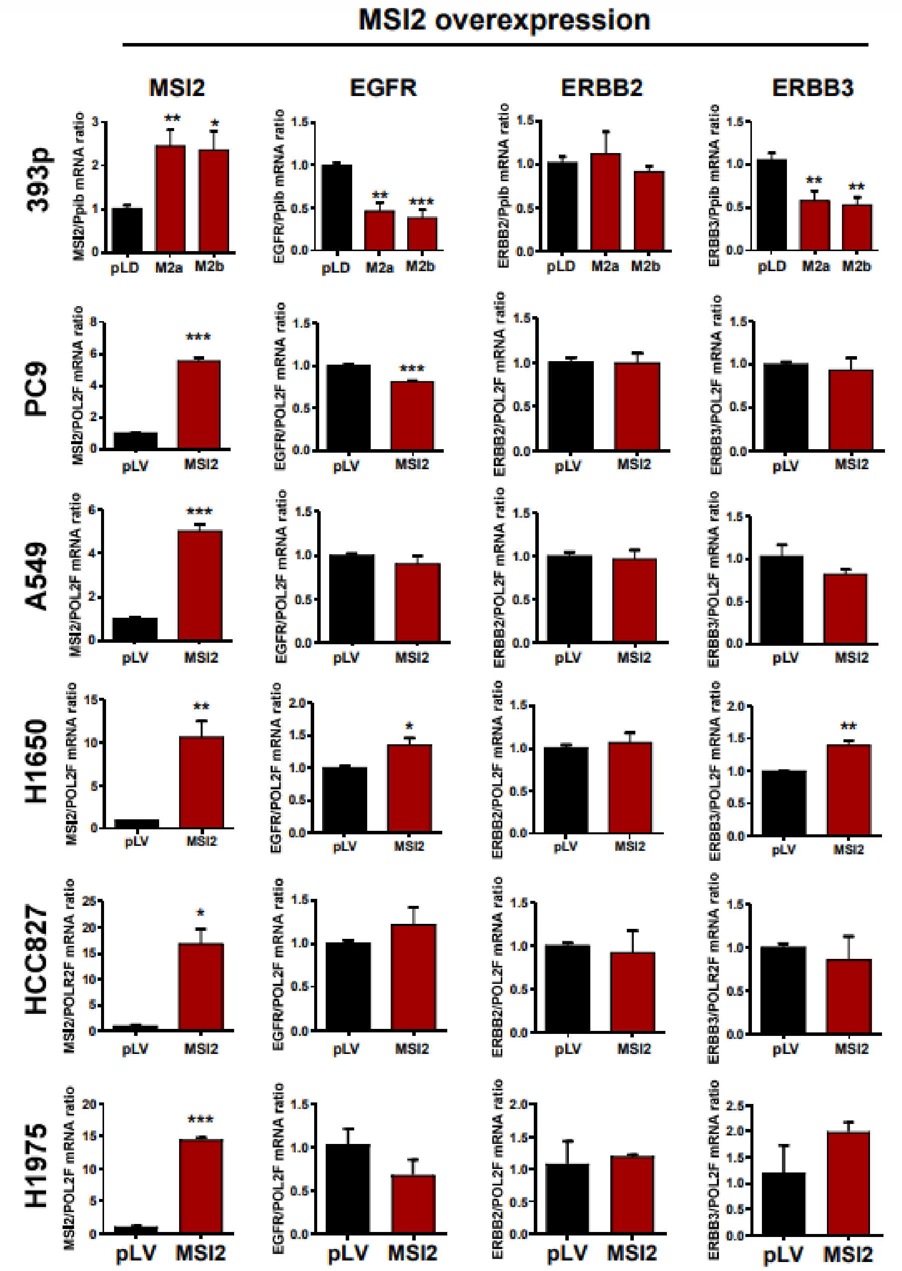


**Supplementary Figure S4**. **Consequences of MSI2 overexpression on mRNA expression of ERBB genes in NSCLC cell line models.** Quantitative RT-PCR of mRNA collected from indicated cell lines, stably overexpressing MSI2 (MSI2) in two KRAS$^{mut}$ (344SQ and A549) and three EGFR$^{mut}$ (H1650, HCC827, and PC9) NSCLC cell lines. Negative controls include pLV-CMV-puro (pLV) and pLenti63/V5 DEST (pLD) empty vector. Data are normalized to *Ppib*, *POLR2F*, or *IPO8*, as noted. Relative quantification (RQ) of gene expression was performed using $2^{-\Delta\Delta Ct}$ method. Data are presented as average RQ means of three independent qRT-PCR experiments. Error bars represented by SEM. Statistical analysis was performed using unpaired two tailed t-test. *, $p<0.05$, **, $p<0.01$, ***, $p<0.001$ for all graphs.

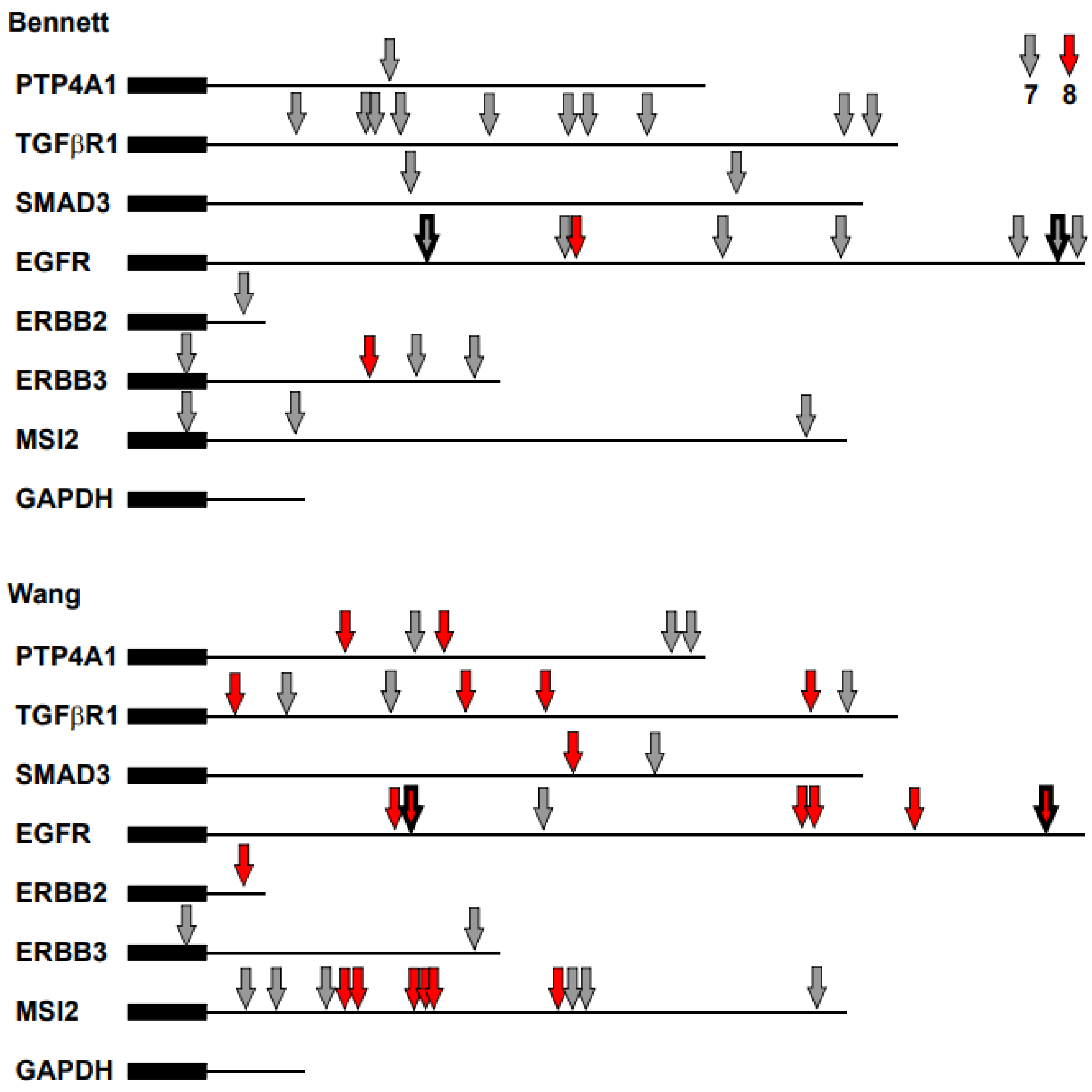


**Supplementary Figure S5. MSI2 consensus binding sites on human mRNA.** Location of consensus binding sites for Musashi proteins in the noted human genes, as defined from studies by Bennett et al(18), and Wang et al (19). Coding sequences are represented by thick lines; 3' untranslated regions by thin line. 7- or 8-bp consensus sequences are indicated by arrows. Thick arrows indicate identical consensus sequences identified simultaneously by Wang and Bennett studies. Shorter consensus sequences are not indicated. Human genome sequences for *EGFR* were obtained from the UCSC Human Gene Sorter December 2013 (GRCh38/hg38) assembly; for genes with multiple transcripts or variations of various lengths, the three sequences with the most total base pairs, including 5' UTR exons, CDS Exons, and 3' UTR exons, were analyzed.

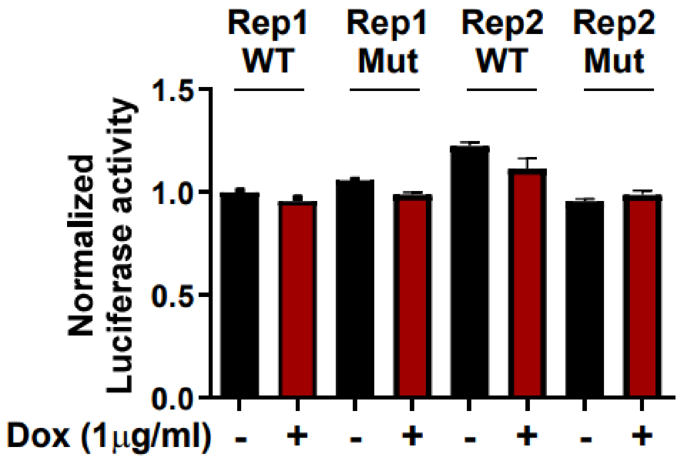


**Supplementary Figure S6. Reporter analysis by DualGlo Luciferase assay.** PC9 cells stably expressing Doxycycline-inducible MSI2 shRNA (sh1 targeting sequence) were transfected with Firefly luciferase MSI2 reporter constructs (reporter 1 (Rep1 WT) and reporter 2 (Rep2 WT)) or their analogs with mutated MSI2 binding sites (Rep1 Mut and Rep2 Mut) were transfected concomitantly with Renilla luciferase vector phRL-TK in triplicates. 24 hours post-transfection 1μg/ml of Doxycycline (Dox+) was added to control wells (negative control - Dox- cells without induction of shRNA expression). Dual Glo Luciferase assay was performed 48 hours after stimulation with Doxycycline. Data presented as normalized Firefly to Renilla Luciferase activity. Error bars represented by SEM.

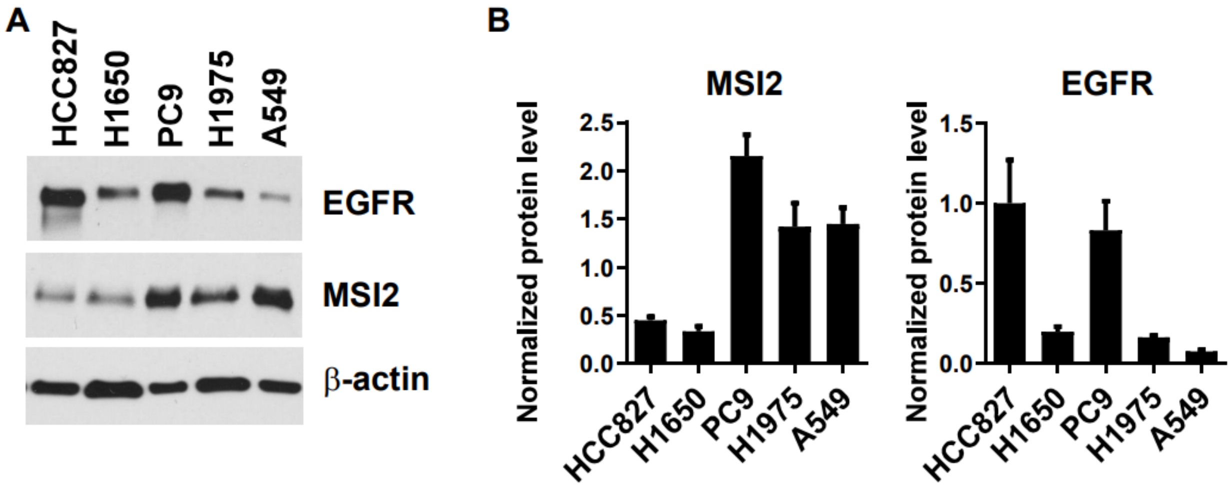


**Supplementary figure S7. Basal MSI2 and EGFR expression levels in 5 tested NSCLC cell lines. A.** Representative western blot analysis of MSI2 and EGFR, protein levels. **B.** Quantification of Western blot data. Normalized quantification of Western blot data is from at least three independent experiments by Image J software. Error bars represented by SEM.

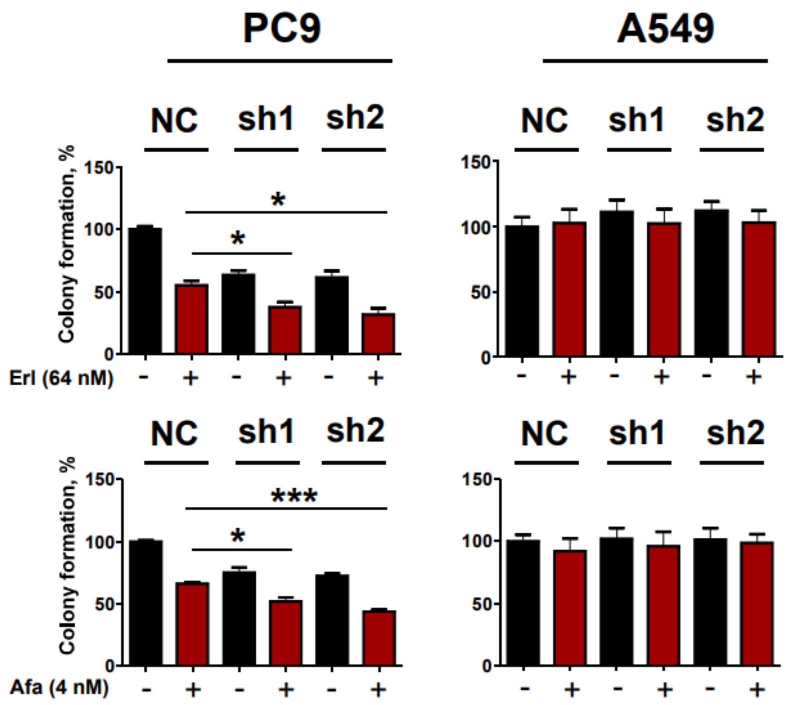


**Supplementary Figure S8.** Clonogenic survival of PC9 and A549 cell line derivatives expressing doxycycline-inducible anti-MSI2 shRNAs (sh1 and sh2) or negative control shRNA (NC) cultured in complete medium in presence of 1μg/ml of Doxycycline with indicated concentrations of erlotinib (Erl) or afatinib (Afa) for 1 week. Data presented represent the average of three independent experiments. Error bars represented by SEM. Statistical analysis was performed using unpaired two tailed t-test. *, $p<0.05$, **, $p<0.01$, ***, $p<0.001$ for all graphs.

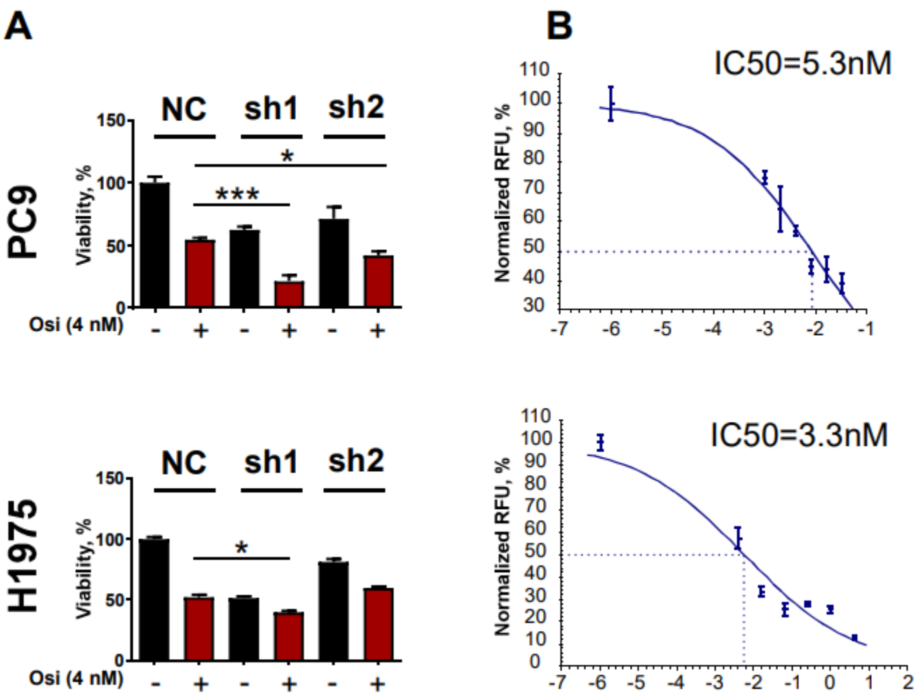


**Supplementary Figure S9. MSI2 knockdown increases the sensitivity of EGFR mutant cell lines to osimertinib treatment. A.** EGFR$^{mut}$ (PC9 and H1975) cell line derivatives expressing doxycycline-inducible anti-MSI2 shRNAs (sh1 and sh2) or negative control (NC) cells were incubated in complete medium in presence of 1μg/ml of doxycycline at the indicated concentrations of osimertinib (Osi) for 96 hours, then viability measured by CTB Assay. Data presented represent the average of three independent experiments. **B.** IC50 curves for viability of cell lines measured by CTB assay following 96 hours treatment with osimertinib. Representative data of one of three independent experiments are presented. Error bars represented by SEM. Statistical analysis was performed using unpaired two tailed t-test. *, p<0.05, **, p<0.01, ***, p<0.001 for all graphs.

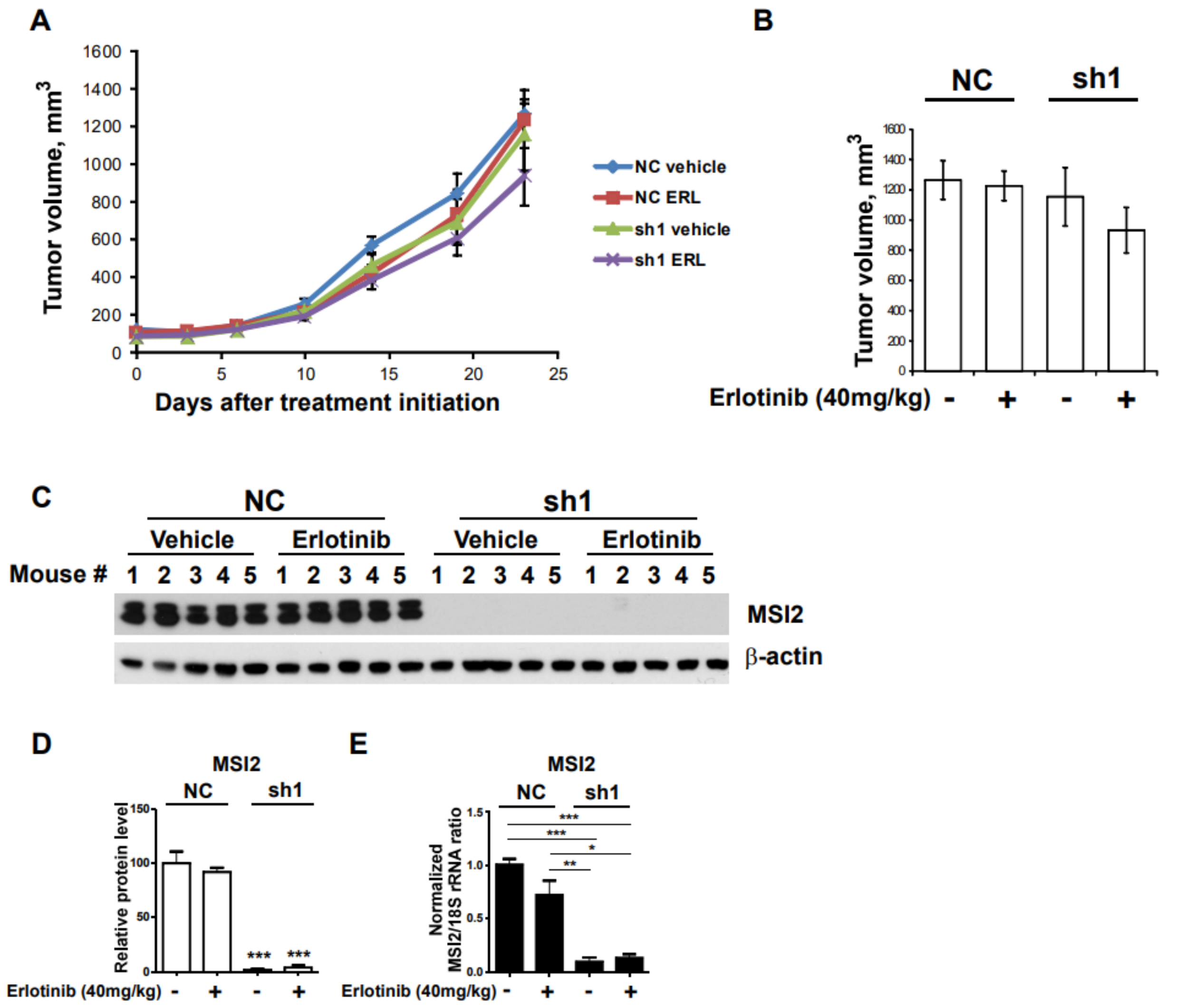


**Supplementary Figure S10. MSI2 knockdown does not increase the sensitivity of EGFR mutant xenograft tumors to erlotinib treatment. A.** Growth curve of subcutaneous xenografts A549 cells stably expressing lentiviral vector as negative control (NC) or shRNA to MSI2 (sh1), and treated with vehicle or erlotinib (ERL) for 24 days. N= 5/group. **B.** Quantification of tumors at endpoint of experiment in **A**. **C.** Western blot analysis of MSI2, protein levels from treated tumors. **D.** Quantification of Western blot data from **C**; data normalized to β-actin. **E**. Quantitative RT-PCR of mRNA collected from indicated xenograft tumors at the end of experiments. Negative controls are denoted NC. Data are normalized to 18S rRNA, as noted. Relative quantification (RQ) of gene expression was performed using $2^{-\Delta\Delta Ct}$ method. Data are presented as normalized average RQ means in each group (n=5) of animals. In all graphs, error bars represented by SEM. Statistical analysis was performed using unpaired two tailed t-test. *, p<0.05, **, p<0.01, ***, p<0.001 for all graphs.

Supp Table S1. Table of consensus MSI2 binding site sequences, Benett et al motifs

| | | **Bennett Motifs: Human Genes** | | | | | | | | | | | | | | | |
|---|---|---|---|---|---|---|---|---|---|---|---|---|---|---|---|---|---|
| | | **PTP4A1** | | **TGFßR1** | | **SMAD3** | | **EGFR** | | **HER2** | | **HER3** | | **MSI2** | | **GAPDH** | |
| **Motifs** | **Translated** | **3'** | **CDS** | **3'** | **CDS** | **3'** | **CDS** | **3'** | **CDS** | **3'** | **CDS** | **3'** | **CDS** | **3'** | **CDS** | **3'** | **CDS** |
| uuuagaa | tttagaa | | | 2 | | | | 2 | | | | 1 | | | | | |
| uguagca | tgtagca | | | 1 | | | | 3 | | | | | | | | | |
| ucuagcu | tctagct | | | 1 | | 1 | | 1 | | | | | | | | | |
| uguagga | tgtagga | | | 1 | | 1 | | 1 | | | | | 1 | | | | |
| auuagag | attagag | | | 2 | | | | 1 | | | | 1 | | | 1 | | |
| uaguag | tagtag + t/a | 1 | | 1 | | | | | | | | | | | | | |
| uuaguu | ttagtt | 3 | | 1 | | 2 | | 3 | | 1 | | | | | | | |
| uuagua | ttagta | 4 | | 2 | 1 | 1 | | | | | | | | 1 | | | |
| uuguaguu | ttgtagtt | | | | | | | 1 | | | | | | | | | |
| uuagu | ttagt | | | | | 4 | | | | | | 1 | | 4 | | | |
| uagu | tagt + a | | | | | | | | 2 | 1 | | 3 | | 2 | | | |
| uaguu | tagtt | | | | | | | | | | | | | 2 | | | |
| auagu | atagt | | | | | | | | | | | | | 3 | | | |
| guag | gtagta | | | | | 1 | | | | | | | | 1 | | | |
| uaguau | tagtat | | | | | 2 | | 2 | | | | | | | | | |
| TOTALS | | 8 | 0 | 11 | 1 | 12 | 0 | | | 2 | | 6 | 1 | 13 | 1 | 0 | 0 |

| | Bennett Motifs: Mouse Genes | | | | | | | | | | | | | | | | |
|---|---|---|---|---|---|---|---|---|---|---|---|---|---|---|---|---|---|
| | | Ptp4a1 | | Tgfbr1 | | Smad3 | | Egfr | | Her2 | | Her3 | | Msi2 | | Gapdh | |
| **Motifs** | **Translated** | **3'** | **CDS** | **3'** | **CDS** | **3'** | **CDS** | **3'** | **CDS** | **3'** | **CDS** | **3'** | **CDS** | **3'** | **CDS** | **3'** | **CDS** |
| uuuagaa | tttagaa | 2 | | 1 | | 1 | | | | | | | | 1 | | | |
| uguagca | tgtagca | | | 1 | | | | 1 | | | | | | | | | |
| ucuagcu | tctagct | | | | | 1 | | | | | | | | 1 | | | |
| uguagga | tgtagga | | | 1 | | 2 | | | | | | 1 | 1 | | | | |
| auuagag | attagag | | | 1 | | 1 | | | | | | | | | 1 | | |
| uaguag | tagtag + t/a | | | 1 | | | | | | | | | | | | | |
| uuaguu | ttagtt | 1 | | 1 | | | | | | | | 1 | | | | | |
| uuagua | ttagta | 4 | | 1 | | | | | | | | | | 4 | | | |
| uuguaguu | ttgtagtt | | | | | | | | | | | | | | | | |
| uuagu | ttagt | 1 | | 4 | 1 | 1 | | 1 | | 1 | | 1 | 1 | 2 | | | |
| uagu | tagt + a | | | 1 | | | | 1 | | | | | | 2 | | | |
| uaguu | tagtt | 1 | | | 1 | | | 2 | 1 | | | 1 | 1 | 1 | | | |
| auagu | atagt | 5 | 1 | 5 | 1 | 1 | | | 1 | | 2 | 1 | 1 | 3 | | | |
| guag | gtagta | | | 3 | | | | | | | | | | | | | |
| uaguau | tagtat | | | | | | | | | | | | | | | | |
| TOTALS | | 14 | 1 | 20 | 3 | 7 | 0 | 5 | 2 | 1 | 2 | 5 | 4 | 14 | 1 | 0 | 0 |

3' = 3' mRNA
CDS = Coding sequence

**Supplementary Table S1**. **Consensus sites for MSI2 binding defined by Bennett et al (1)**. Each defined motif is listed as mRNA and translated sequence. Number of detected motifs fount in the 3' UTR, or within the coding sequence (CDS), is noted for each indicated gene.

Supp Table S2. Consensus MSI2 binding site sequences, defined by Wang et al.

| | | Human: Wang Motifs | | | | | | | | | | | | | | | |
|---|---|---|---|---|---|---|---|---|---|---|---|---|---|---|---|---|---|
| | | PTP4A1 | | TGFßR1 | | SMAD3 | | EGFR | | HER2 | | HER3 | | MSI2 | | GAPDH | |
| **Motifs** | **Translated** | **3'** | **CDS** | **3'** | **CDS** | **3'** | **CDS** | **3'** | **CDS** | **3'** | **CDS** | **3'** | **CDS** | **3'** | **CDS** | **3'** | **CDS** |
| accuuuuu | accttttt | | | 1 | | | | 1 | | | | | | | | | |
| uuuuagaa | ttttagaa | | | 1 | | | | 3 | | | | | 1 | | | | |
| uuuuuaaa | tttttaaa | 3 | | 3 | | 2 | | 2 | | 1 | | | | 6 | | | |
| uagagu | tagagt | 1 | | 4 | | 3 | | | | | | | | | | | |
| uagagc | tagagc | | | 1 | | 1 | | | | | | | 2 | 1 | | | |
| ucuagu | tctagt | | | | | 1 | | 1 | | | | | | 3 | | | |
| ucuagc | tctagc | | | 1 | | 2 | | 1 | | | | | | 2 | | | |
| ugccaa | tgccaa | 1 | | 1 | 2 | | | | 3 | | 3 | 1 | 5 | | | | 3 |
| ugcuaa | tgctaa | 2 | | | | | | 3 | | | | | | 2 | | | |
| uaugugu | tatgtgt | 1 | | 1 | | | | 1 | | | | | 1 | | | | |
| ugugugu | tgtgtgt | 2 | | | | 1 | | | | | | | | 1 | | | |
| ucugugu | tctgtgt | | | 1 | | 1 | | 1 | | | | | | 2 | | | |
| gugucu | gtgtct | 1 | | 1 | | 4 | 1 | 2 | | 1 | 1 | 1 | 2 | | | | |
| uagaagu | tagaagt | | | 1 | | | | | | | | | | 2 | | | |
| uaggucu | taggtct | | | | | | | | | | | | | | | | |
| uagaag | tagaag | 2 | | 2 | | | | 2 | | | | | | 2 | | | |
| TOTALS | | 13 | 0 | 18 | 2 | 15 | 1 | 17 | 3 | 2 | 4 | 2 | 11 | 21 | 0 | 0 | 3 |

| | | Mouse: Wang Motifs | | | | | | | | | | | | | | | |
|---|---|---|---|---|---|---|---|---|---|---|---|---|---|---|---|---|---|
| | | **PTP4A1** | | **TGFßR1** | | **SMAD3** | | **EGFR** | | **HER2** | | **HER3** | | **MSI2** | | **GAPDH** | |
| **Motifs** | **Translated** | **3'** | **CDS** | **3'** | **CDS** | **3'** | **CDS** | **3'** | **CDS** | **3'** | **CDS** | **3'** | **CDS** | **3'** | **CDS** | **3'** | **CDS** |
| accuuuuu | accttttt | | | | 1 | | | | | | | | | | | | |
| uuuuagaa | ttttagaa | | | | | 1 | | | | | | | | | | | |
| uuuuuaaa | tttttaaa | | | 3 | | | | | | 1 | | | | 9 | | | |
| uagagu | tagagt | 1 | | 1 | | 2 | | | | | | | | | | | |
| uagagc | tagagc | | | | | 2 | | 2 | | | | | | | | | |
| ucuagu | tctagt | | | | | | | 1 | | | | 1 | | 2 | | | |
| ucuagc | tctagc | 1 | | | | 1 | | | | | | | 1 | 2 | | | |
| ugccaa | tgccaa | 2 | | 3 | 1 | | | | 4 | | 1 | | 3 | | | | 3 |
| ugcuaa | tgctaa | | | 1 | | | | | | | | | | 2 | | | |
| uaugugu | tatgtgt | | | 1 | 1 | 1 | | 1 | | | 1 | 1 | | | | | |
| ugugugu | tgtgtgt | | | | | | 1 | | | | | | | 1 | | | |
| ucugugu | tctgtgt | | | 1 | | | | | | | | | 1 | 1 | | | |
| gugucu | gtgtct | 2 | | 2 | | 3 | | 1 | 1 | | 1 | 2 | 3 | 1 | | | |
| uagaagu | tagaagt | 1 | | | | | | | | | | | | 2 | | | |
| uaggucu | taggtct | | | | | | | | | | | | 1 | | | | |
| uagaag | tagaag | 1 | | | | 2 | | | | | | | 2 | | | | |
| TOTALS | | 8 | 0 | 12 | 3 | 12 | 1 | 5 | 5 | 1 | 3 | 4 | 11 | 20 | 0 | 0 | 3 |

3' = 3' mRNA
CDS = Coding sequence

**Supplementary Table S2**. **Consensus sites for MSI2 binding defined by Wang et al (2)**. Each defined motif is listed as mRNA and translated sequence. Number of detected motifs fount in the 3' UTR, or within the coding sequence (CDS), is noted for each indicated gene.

| ssRNA-oligo name | Sequence |
|---|---|
| EGFR Oligo 1 wt | 5`-gcagauguuuuagaaggaaaaa |
| EGFR Oligo 1 mut | 5`-gcagaggccccagcaggaaaaa |
| EGFR Oligo 2 wt | 5`-uaacuucguuuuagaaacauucaag |
| EGFR Oligo 2 mut | 5`-uaacuccgccccagcaacauucaag |
| Positive Control oligo | 5`-guaguaguaguagua |
| Negative Control oligo | 5`-ggucccaagccaagccuuaagu |

**Supplementary Table S3. Sequences of single stranded RNA (ssRNA) oligos used in RNA-EMSA analysis.**

| Gender | % |
|---|---|
| Male | 50 |
| Female | 50 |
| **Age at diagnosis** | **Years** |
| Mean | 59 |
| Min | 44 |
| Max | 72 |
| SD | 8.6 |
| **Histology** | **%** |
| Adenocarcinoma, NOS | 100 |
| **Overall stage** | **%** |
| 1A | 4.5 |
| 2A | 41 |
| 2B | 4.5 |
| 3A | 9 |
| 3B | 27.3 |
| 4 | 13.7 |
| **T stage** | **%** |
| 1 | 9 |
| 2 | 59 |
| 3 | 23 |
| 4 | 9 |
| **Lymph Nodes** | **%** |
| 0 | 54.5 |
| 1 | 9.1 |
| 2 | 9.1 |
| 3 | 18.2 |
| x | 9.1 |
| **M stage** | **%** |
| 0 | 77.3 |
| 1 | 13.7 |
| x | 9 |
| **Grade** | **%** |
| Moderately differentiated | 60 |
| Poor differentiated | 40 |
| **EGFR mutation** | **%** |
| T790M | 4.5 |
| L858R | 50 |
| Ex19del | 45.5 |

**Supplementary Table S4. Clinical characteristics of EGFR$^{mut}$ NSCLC specimens from Rostov Research Institute Human Tissue Repository Facility (HTRF).** The tissue microarray used for IHC contained specimens from 22 patients with characteristics noted in the table.

| | ssDNA oligos |
|---|---|
| Mouse, m1 | Fw: 5`-CCGGCCCAACTTTGTGGCAACCTATCTCGAGATAGGTTGCCACAAAGTTGGGTTTTT |
| | Rev: 5`-ATTAAAAACCCAACTTTGTGGCAACCTATCTCGAGATAGGTTGCCACAAAGTTGGG |
| Mouse, m2 | Fw: 5`-CCGGCGTAGGAGGATTGTCTGCAAACTCGAGTTTGCAGACAATCCTCCTACGTTTTT |
| | Rev: 5`-AATTAAAAACGTAGGAGGATTGTCTGCAAACTCGAGTTTGCAGACAATCCTCCTACG |
| Human, sh1 | Fw: 5`- CCGGGTGGAAGATGTAAAGCAATATCTCGAGATATTGCTTTACATCTTCCACTTTTT |
| | Rev: 5`-AATTAAAAAGTGGAAGATGTAAAGCAATATCTCGAGATATTGCTTTACATCTTCCAC |
| Human, sh2 | Fw: 5`-CCGGCCCAACTTCGTGGCGACCTATCTCGAGATAGGTCGCCACGAAGTTGGGTTTTT |
| | Rev: 5`-AATTAAAAACCCAACTTCGTGGCGACCTATCTCGAGATAGGTCGCCACGAAGTTGGG |

**Supplementary Table S5. DNA oligonucleotides used for construction of shRNA vectors.** Table lists single stranded DNA (ssDNA) oligos used for generation of Tet-pLKO vectors expressing specific shRNAs, used for lentiviral infection and selection of stable cell lines. MSI2 targeting sequences are underscored.

| Name | Vector | Type of insert | Type of expression | Cell line origin |
|---|---|---|---|---|
| A549 NC | Tet-pLKO-puro | Empty | no | Human |
| A549 sh1 | Tet-pLKO-sh1-puro | MSI2-shRNA1 | Inducible | Human |
| A549 sh2 | Tet-pLKO-sh2-puro | MSI2-shRNA2 | Inducible | Human |
| A549 pLV | pLV-CMV-puro | Empty | no | Human |
| A549 MSI2 | pLV-CMV-MSI2-puro | MSI2 ORF | Constitutive | Human |
| PC9 NC | Tet-pLKO-puro | Empty | no | Human |
| PC9 sh1 | Tet-pLKO-sh1-puro | MSI2-shRNA1 | Inducible | Human |
| PC9 sh2 | Tet-pLKO-sh2-puro | MSI2-shRNA2 | Inducible | Human |
| PC9 pLV | pLV-CMV-puro | Empty | no | Human |
| PC9 MSI2 | pLV-CMV-MSI2-puro | MSI2 ORF | Constitutive | Human |
| Hcc827 NC | Tet-pLKO-puro | Empty | no | Human |
| Hcc827 sh1 | Tet-pLKO-sh1-puro | MSI2-shRNA1 | Inducible | Human |
| Hcc827 sh2 | Tet-pLKO-sh2-puro | MSI2-shRNA2 | Inducible | Human |
| Hcc827 pLV | pLV-CMV-puro | Empty | no | Human |
| Hcc827 MSI2 | pLV-CMV-MSI2-puro | MSI2 ORF | Constitutive | Human |
| 344SQ pLKO | pLKO.1-puro | Empty | no | Mouse |
| 344SQ m1 | pLKO-m1-puro | MSI2-shRNA1 | Constitutive | Mouse |
| 344SQ m2 | pLKO-m2-puro | MSI2-shRNA2 | Constitutive | Mouse |
| 393p pLD | pLenti63/V5 DEST | Empty | Constitutive | Mouse |
| 393p M2a | pLenti63/V5 DEST | MSI2 ORF | Constitutive | Mouse |
| 393p M2b | pLenti63/V5 DEST | MSI2 ORF | Constitutive | Mouse |

**Supplementary Table S6. Stable cell lines used in study.** List of cell line derivatives used in the study. For human cell lines, the lentiviral vectors Tet-pLKO-puro (Addgene, Plasmid #21915) was used for inducible expression of shRNAs. For murine cell lines, the lentiviral vectors pLKO.1-puro (Addgene, Plasmid #8453) was used for stable expression of shRNAs. For human cell lines, the pLV-CMV-puro vector (a kind gift from Dr. A. Ivanov, West Virginia University) was used for stable expression of the MSI2 cDNA. For murine cell lines, the pLenti63/V5 DEST vector (Thermo Fisher Scientific, Waltham, MA) was used for expression of the MSI2 cDNA.

| siRNA Catalog Number | Gene Symbol | Sequence |
|---|---|---|
| SI04236652 and SI04285834 | Human MSI2 "-h1" (Mixture 2) | ATGAGAGATCCCACTACGAAA and CUGGAUUGGUCAUCAGAUU |
| SI04312665 and SI04375847 | Human MSI2 "-h2" (Mixture 1) | TCCCAACTTCGTGGCGACCTA and CCAGATAGCCTTAGAGACTAT |
| SI04465426 and SI04958079 | Mouse Msi2 "-m1" (Mixture 2) | TTCCAAGACGATTGACCCAAA and GCAAGTGTAGATAAAGTATTA |
| SI04958086 and SI04958093 | Mouse Msi2 "-m2" (Mixture 1) | ATGAGAGATCCCACAACGAAA and CCAGATAGCCTTAGAGACTAT |

**Supplementary Table S7. siRNAs targeting human and murine MSI2**. siRNA reference numbers are from Qiagen (Frederick, MD).

| Gene symbol (H)-human; (M)-mouse | SYBR Green | Taqman Life Technologies |
|---|---|---|
| *PTP4A1* (H) | Fw: 5`-ATCCAACCAATGCGACCTTA<br>Rev: 5`-AAGGCCAATCAAGAACATGG | |
| *GAPDH* (H) | Fw: 5`-TGCACCACCAACTGCTTAGC<br>Rev: 5`-GGCATGGACTGTGGTCATGAG | |
| *TGFBR1* (H) | | Hs00610320_m1 |
| *EGFR* (H) | | Hs01076092_m1 |
| *ERBB2* (H) | Fw: 5`-GTGACTGCCTGTCCCTACAACTA<br>Rev: 5`-TGTGTTCCATCCTCTGCTGTC | |
| *ERBB3* (H) | Fw: 5`-TGCTATACAGTGAGGCCAAGACTC<br>Rev: 5`-CAACTCCCAAACTGTCACACCATA | |
| *SMAD3* (H) | Fw: 5`-CCAGCACATAATAACTTGGACCT<br>Rev: 5`-GATGTGTCTCCGTGTCAGCTC | |
| *MSI2* (H) | Fw: 5`-GGTCATGAGAGATCCCACTACG<br>Rev: 5`-TCTACACTTGCTGGGTCTGC | |
| *POLR2F* (H) | | Fw: 5`-TGCCATGAAGGAACTCAAGG<br>Rev: 5`-TCATAGCTCCCATCTGGCAG<br>Probe: 6fam-CCCCATCATCATTCGCCGTTACC-bhq1 |
| *18S rRNA* (H) | | Fw: 5` GCTCTTTCTCGATTCCGT<br>Rev: 5`- CCAGAGTCTCGTTCGTTATC<br>Probe: 6fam-TTCTTAGTTGGTGGAGCGATTTGT-Iowa blackFQ |
| *IPO8* (H) | | Hs00183533_m1 |
| *Ppib* (M) | | Mm00478295_m1 |
| *Msi2* (M) | Fw: 5`-AGCAGTATTTCGAGCAGTTTGGCA<br>Rev: 5`-TGTCGAACATCAGCATCGCATCC | |
| *Egfr* (M) | | Mm00433023_m1 |
| *Erbb2* (M) | Fw: 5`-GTGTGGAGGAGTGCCGAGTAT<br>Rev: 5`-ACACTGGTCAGCCTCCGATC | |
| *Erbb3* (M) | | Mm00695835_m1 |

**Supplementary Table S8. Primers used for RT-PCR to quantify gene expression.** List of primers used for SYBR Green assays, and TaqMan gene expression assays, used for qRT-PCR analysis of gene expression and RNA immunoprecipitation.

**References.**

1. Bennett CG, Riemondy K, Chapnick DA, Bunker E, Liu X, Kuersten S*, et al.* Genome-wide analysis of Musashi-2 targets reveals novel functions in governing epithelial cell migration. Nucleic Acids Res **2016**;44(8):3788-800 doi 10.1093/nar/gkw207.
2. Wang S, Li N, Yousefi M, Nakauka-Ddamba A, Li F, Parada K*, et al.* Transformation of the intestinal epithelium by the MSI2 RNA-binding protein. Nat Commun **2015**;6:6517 doi 10.1038/ncomms7517.
